# Viral encephalitis and seizures cause rapid depletion of neuronal progenitor cells and alter neurogenesis in the adult mouse dentate gyrus

**DOI:** 10.3389/fncel.2024.1528918

**Published:** 2025-01-14

**Authors:** Alberto Pauletti, Polina Gurlo, Edna Weiß, Ana Beatriz DePaula-Silva, Karen S. Wilcox, Sonja Bröer

**Affiliations:** ^1^School of Veterinary Medicine, Institute of Pharmacology and Toxicology, Freie Universität Berlin, Berlin, Germany; ^2^Department of Pharmacology and Toxicology, University of Utah, Salt Lake City, UT, United States

**Keywords:** virus infection, TMEV, seizure, neurogenesis, progenitor, cell proliferation, epilepsy

## Abstract

Infections impacting the central nervous system (CNS) constitute a substantial predisposing factor for the emergence of epileptic seizures. Given that epilepsy conventionally correlates with hippocampal sclerosis and neuronal degeneration, a potentially innovative avenue for therapeutic intervention involves fostering adult neurogenesis, a process primarily occurring within the subgranular zone of the dentate gyrus (DG) through the differentiation of neural stem cells (NSC). While experimental seizures induced by chemoconvulsants or electrical stimulation transiently enhance neurogenesis, the effects of encephalitis and the resultant virus-induced seizures remain inadequately understood. Thus, this study employed the Theiler's Murine Encephalomyelitis Virus (TMEV) model of virus-induced seizures in adult C57BL/6J mice to investigate the impact of infection-induced seizures on neurogenesis at three distinct time points [3, 7, and 14 days post-infection (dpi)]. Immunohistochemical analysis revealed a reduction in the overall number of proliferating cells post-infection. More notably, the specific cell types exhibiting proliferation diverged between TMEV and control (CTR) mice: (1) Neuronal progenitors (doublecortin, DCX^+^) were almost entirely absent at 3 dpi in the dorsal DG. They resumed proliferation at 14 dpi, but, did not recover to CTR levels, and displayed aberrant migration patterns. (2) The number of proliferating NSCs significantly decreased within the dorsal DG of TMEV mice at 14 dpi compared to CTR, while (3) a heightened population of proliferating astrocytes was observed. Most observed changes were not different between seizing and non-seizing infected mice. In summary, our findings demonstrate that viral infection rapidly depletes neuronal progenitor cells and causes aberrant migration of the remaining ones, potentially contributing to hyperexcitability. Additionally, the increased differentiation toward glial cell fates in infected mice emerges as a possible additional pro-epileptogenic mechanism.

## Graphical Abstract

Infection-Induced Seizures Alter Neurogenesis in the TMEV Model. This study employed the Theiler's Murine Encephalomyelitis Virus (TMEV) model to investigate the effects of virus-induced seizures on neurogenesis at 3, 7, and 14 days post-infection (dpi) in adult C57BL/6J mice. Immunohistochemical analysis revealed a reduction in proliferating cells post-infection. Notably, neuronal progenitors (DCX+) were nearly absent at 3 dpi, resumed proliferation by 14 dpi, but did not reach control (CTR) levels and displayed abnormal migration. Additionally, the number of proliferating neural stem cells (NSCs) decreased significantly in TMEV mice at 14 dpi, while a higher population of proliferating astrocytes was observed. Most changes were similar between seizing and non-seizing infected mice. 
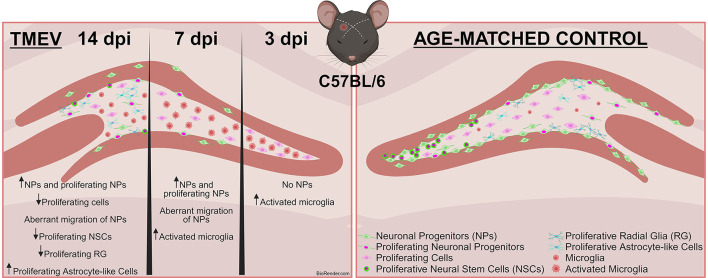

## 1 Introduction

Epilepsy is a profoundly debilitating neurological disorder characterized by recurrent unprovoked seizures, afflicting over 70 million people worldwide. Currently available antiseizure drugs offer symptomatic seizure control but are often accompanied by adverse effects and regrettably demonstrate inefficacy in nearly one-third of the patients (Löscher et al., 2020), underscoring the imperative for innovative therapeutic approaches. One such pioneering intervention is regenerative medicine which could offer more than just symptomatically balancing inhibition and excitation in the epileptic brain: Replacing lost inhibitory neurons via transplantation of gamma-aminobutyric acid (GABA)-ergic interneurons derived from human stem cells has shown promising results in experimental epilepsy models (Bershteyn et al., 2023; Cunningham et al., 2014; Upadhya et al., 2019; Waloschková et al., 2021). However, critical concerns regarding tumorigenicity and invasive delivery into the patient's brain must be addressed (Yasuhara et al., 2017). Alternatively, targeting endogenous repair mechanisms, such as the birth of new neurons (adult neurogenesis), to counteract progressive neuron loss and aberrant network connectivity, presents a promising therapeutic strategy. In fact, recent investigations (Jessberger and Parent, 2015) have highlighted the complex and important relationship between epileptic disease progression and adult neurogenesis, which introduces a largely unexplored delicate equilibrium between the brain regenerative processes and pathological outcomes.

Neurogenesis, the process of generating and integrating new neurons into specific brain regions, has undergone a paradigm shift from being largely attributed to early brain development to being acknowledged as an ongoing phenomenon in specific brain neurogenic niches during adulthood (Gross, 2000; Hussain et al., 2024; Wang et al., 2022). These niches, namely the dentate gyrus (DG) in the hippocampus and the subventricular zone (SVZ) of the lateral ventricles, have the capacity to generate new neurons (Abbott and Nigussie, 2020; Kempermann et al., 2018; Schlessinger et al., 1975). In fact, despite the inherent constraints on neuronal regeneration within the mature brain, a notable 6% of cellular constituents within the DG are identified as adult-born neurons (Cameron and McKay, 2001), and are known to persist long term, as substantiated by rodent studies conducted by Dayer et al. (2003). Key to the dynamics of adult neurogenesis are the NSCs within the DG, characterized by their radial morphology. NSCs mature into neurons, migrate to their appropriate locations within existing neural circuits, and establish functional connections. Adult-born neurons must correctly and precisely integrate into the already existing neural networks to be able to contribute to the physiological brain function such as cognitive abilities, including learning and memory (Bao and Song, 2018; Christian et al., 2014; Deng et al., 2010; Sahay and Hen, 2007). Emerging evidence unveiled the unique role of immature adult-born neurons in modulating neural networks. These immature neurons exhibit heightened excitability and plasticity, attributes that enable them to differentially influence mature neuron firing, synchronization, and network oscillations, thus contributing to cognitive processes (Bao and Song, 2018; Song et al., 2012). Moreover, their connections within the hippocampal circuitry and broader connectivity to other brain regions underscore their significance in regulating vast neural dynamics (Bao and Song, 2018; Barbosa et al., 2015; Lugert et al., 2010; Pilz et al., 2018).

Given its importance, the process of adult neurogenesis is under stringent regulation by a diverse array of intrinsic and extrinsic factors: Environmental enrichment, physical activity, learning experiences, and exposure to novel stimuli enhance neurogenesis, whereas stress, aging, and specific neurological pathologies like epilepsy can alter the process. Aberrations in adult neurogenesis have been implicated in a range of cerebral disorders such as major depression, neurodegenerative diseases, and epilepsy (Christian et al., 2014; Kang et al., 2016; Sahay and Hen, 2007; Winner et al., 2011). In fact, extended seizures trigger adult neurogenesis in several animal models, presumably as a compensatory mechanism against neuronal loss (Bengzon et al., 1997; Parent et al., 1997). However, neurons born during and after seizures exhibit structural and functional abnormalities, leading to enduring changes in hippocampal morphology and heightened neuronal hyperexcitability (Jessberger et al., 2007).

Our understanding of (aberrant) adult neurogenesis in epilepsy is primarily based on traditional epilepsy models where seizures are induced chemically or electrically. However, the etiology of epilepsy encompasses an array of central nervous system (CNS) insults, including genetic causes, traumatic brain injuries, brain tumors, and infections (Fisher et al., 2017). Notably, brain inflammation due to cerebral infections (encephalitis) emerges as a pivotal risk factor for acute seizures and subsequent epilepsy onset. Numerous viruses, including herpes simplex virus type-1 (HSV-1), non-polio picornaviruses, Zika virus (ZIKV), West Nile virus (WNV), Japanese encephalitis virus, cytomegalovirus, human herpes virus-6, and SARS-CoV-2, can trigger encephalitis in humans (Bartolini et al., 2019; DePaula-Silva et al., 2021; Ellul et al., 2020; Solomon et al., 2000; Suzuki et al., 2008). Importantly, the repercussions of viral encephalitis extend beyond the acute phase. After virus infection, an initiated tangled response characterized by acute inflammation induces modification of the brain circuitry thereby possibly contributing to the development of epilepsy months to years later (Gerhauser et al., 2019; Libbey et al., 2008; Stewart et al., 2010). This transient provocation of acute seizures first—and the likely occurrence of chronic epilepsy later, propel viral encephalitis into the forefront of scientific inquiry and therapeutic investigation. However, the intersection of infection, neurogenesis, and possible consequent seizure occurrence remains incompletely elucidated. We aim to shed light onto the complex mechanisms governing this relationship and possibly finding new targets for developing innovative therapies.

To investigate the impact of viral infections on adult neurogenesis and its potential implications for epilepsy onset, our study utilized a model of viral encephalitis-induced seizures, the Theiler's Murine Encephalomyelitis Virus (TMEV) model. TMEV's experimental deployment as an epilepsy model is predominantly focused on the assessment of antiseizure drugs (Batot et al., 2022; Metcalf et al., 2022). Recently, it has emerged as a promising model for unveiling various mechanisms underlying virus-induced epilepsy due to its remarkable parallels with human temporal lobe epilepsy (Bröer et al., 2017, 2016; Cusick et al., 2017; DePaula-Silva et al., 2021; Hanak et al., 2019; Howe et al., 2022; Kirkman et al., 2010; Loewen et al., 2016; Sanchez et al., 2019; Waltl et al., 2018a,b). The presented research seeks to start unraveling the temporal dynamics and specific cell types arising from adult-born cells during acute encephalitis, which can be accompanied by seizures, and ends with viral clearance and resolving of symptoms. Thus, in the TMEV model, the acute phase lasts up to 14 days post-infection (dpi).

## 2 Materials and methods

### 2.1 Animal model

Procedures involving animals and their care were conducted in conformity with the institutional guidelines and in compliance with national and international laws and policies [EEC Council Directive 2019/1010, June 5, 2019, EU Directive 2010/63/EU for animal experiments, and the ARRIVE guidelines (Percie du Sert et al., 2020)], as well as authorized by the Institutional Animal Care and Use Committee at the University of Utah (protocol number: 21-11009, Dept. of Pharmacology and Toxicology) or the local government [Berlin State Office for Health and Social Affairs (LAGeSo) Berlin, Germany, permission number G0015/21]. All experiments were designed and planned to minimize the number of animals used and their suffering. Animals were housed at constant temperature (23 ± 1°C) and relative humidity (60 ± 5%) with *ad libitum* access to food and water and maintained on a fixed 12 h light/dark cycle. Each cage was enriched with environmental stimuli, including shelters, nesting materials, and plastic and cardboard rolls. TMEV-infected and perfused brain samples were obtained from male and female adult C57BL/6J mice, kindly provided by Prof. Dr. Karen Wilcox from the College of Pharmacy, University of Utah, USA. Additionally, age-matched, in-house-bred naïve male and female C57BL/6J mice were utilized as control (CTR) animals, alongside Dulbecco's modified eagle Medium (DMEM)-injected animals as a vehicle control (TMEV vehicle group). Statistical analyses confirmed no significant differences between naïve CTR and DMEM vehicle controls, allowing the use of CTR animals as representative of baseline physiological conditions (Supplementary Figures 1A–D). Additionally, given that no statistical differences were observed between male and female mice, data from both sexes were combined when appropriate (filled circles indicate male mice, while hollow circles denote females). Finally, required group sizes were determined *a priori* by power analyses (Faul et al., 2009).

### 2.2 TMEV infection and seizure monitoring

**Figure 1:**
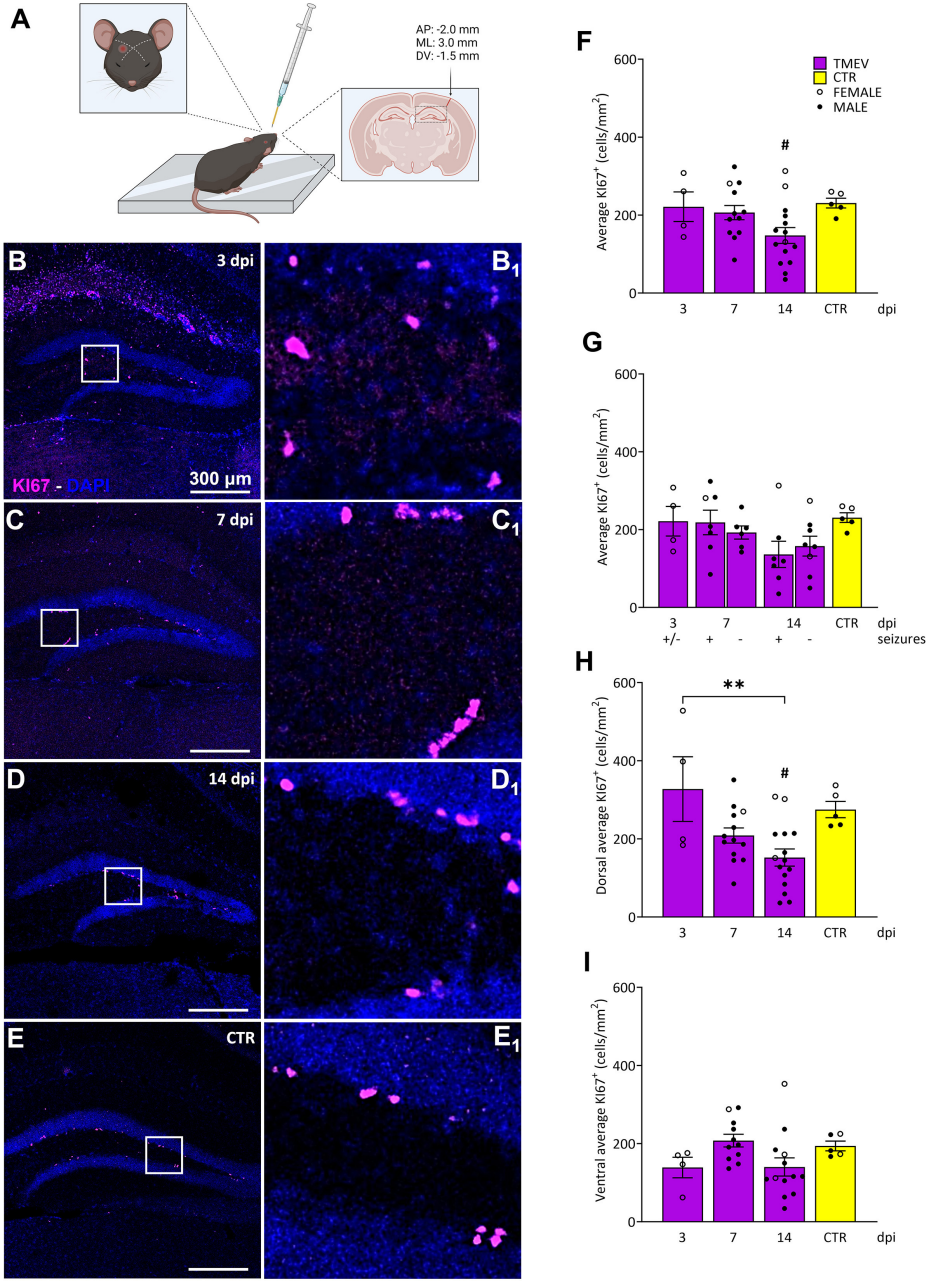
Impact of TMEV CNS infection on DG cell proliferation. **(A)** Schematic illustration depicting the procedure for TMEV injection. Commencing from the left: For administering an injection into the right parietal cortex, the needle is introduced slightly lateral to an imaginary line drawn between the eye and the ear on the contralateral side. The depth control collar is distinctly outlined in yellow to ensure accurate insertion depth. The trajectory of the injection is discernible in the coronal cross-section of the brain, delineated by the arrow. The specified injection site corresponds to the provided coordinates positioned above the arrow. This visual depiction was generated utilizing biorender.com and adapted from Batot et al. (2022). **(B–E)** Representative images showing IHC of KI-67 in C57BL/6J-mouse hippocampal slices at different time points post infection [3 dpi (*n* = 4) **(B)**, 7 dpi (*n* = 13) **(C)**, 14 dpi (*n* = 16) **(D)**, and CTR (*n* = 5) **(E)**]. **(B**_**1**_**–E**_**1**_**)** Insets show magnified views of the corresponding ROIs in **(B–E)**. **(F)** Average KI-67^+^ cell density was analyzed across all the TMEV-infected mice, divided by time point and CTR. A significant reduction in cell proliferation was observed at 14 dpi compared to CTR (14 dpi vs. CTR: #*p* = 0.0270). **(G)** Analyses of seizing (+) and non-seizing (–) mice revealed no significant differences in cell proliferation at 7 and 14 dpi. Mice at 3 dpi were defined as (+/–) due to potential seizure occurrence outside the single observational period before their sacrifice. **(H)** When analyzing the dorsal hippocampi separately, a significant reduction in cell proliferation was detectable at 14 dpi compared to 3 dpi and to CTR (3 dpi vs. 14 dpi: **p = 0.0046, 14 dpi vs. CTR: #*p* = 0.0411). **(I)** No significant differences in cell proliferation were detected in the ventral DG. The scale bar in **(B)** also applies to **(C–E)**. Filled circles indicate male mice, while hollow circles denote females. Data are mean ± SEM, normality was tested using the Shapiro-Wilk test, statistical analyses were performed using Ordinary one-way ANOVA followed by Bonferroni's multiple comparison test. Statistical significance was set at the adjusted *P*-value ≤ 0.05.

Mice were infected using the Daniel's strain (DA) of TMEV in the laboratory of Prof. Dr. Karen Wilcox as detailed in a prior publication (Batot et al., 2022; Figure 1A). Briefly, 7–8 weeks old mice were anesthetized by inhalation of isoflurane and, using a free-hand method of infection that obviated the need for a stereotaxic frame, 20 μL of virus suspension containing 4 × 10^4^ plaque-forming units (PFU) of DA-TMEV, was injected at a depth of 1.5 mm in the right temporal lobe region above the hippocampal formation. Injection coordinates were selected according to stereotaxic injection in relation to bregma [−2.0 (AP); +3.0 (ML); −1.5 (DV)] Paxinos and Franklin (2004). Mice were randomly assigned to three distinct experimental cohorts: 3 (seizure onset), 7 (seizure peak), and 14 dpi (virus clearance and several days after seizures end). Evaluation of the development, intensity, and number of generalized acute seizures was assessed in the laboratory of Prof. Dr. Karen Wilcox and involved monitoring handling-induced behavioral seizures for 1 h twice daily (between 9 AM and 12 PM and between 1 PM and 4 PM), with a minimum 3 h interval between sessions, as outlined by Batot et al. (2022). Generalized motor seizure severity was scored using a modified Racine scale (Racine, 1972; Table 1).

**Table 1:** List of infected mice, seizure scores, and number of seizures (N. of seizures).

**Seizure score**												
		**3 dpi**		**4 dpi**		**5 dpi**		**6 dpi**		**7 dpi**		
**Mouse ID**	**DPI**	**AM**	**PM**	**AM**	**PM**	**AM**	**PM**	**AM**	**PM**	**AM**	**PM**	**N. of seizures**
1	3	5										1
2	3	–										0
3	3	–										0
4	3	–										0
5	7	–	–	–	3	4	4	–	6	–	–	4
6	7	–	–	–	5	–	–	–	–	–	–	1
7	7	–	–	–	–	5	–	6	6	–	–	3
8	7	–	–	–	–	–	5	–	–	–	–	1
9	7	–	–	–	–	–	–	–	–	–		0
10	7	–	–	–	–	–	–	–	–	–		0
11	7	–	–	–	–	–	–	–	–	–		0
12	7	–	–	–	–	–	–	–	–	–		1
13	7	–	–	–	5	–	6	6	6	5		5
14	7	–	–	–	–	–	6	6	6	–		3
15	7	–	–	–	–	–	–	–	–	–		0
16	7	–	–	–	–	–	–	–	–	–		0
17	7	–	3	4	5	6	–	5	–	–		5
18	14	–	–	5	5	5	6	6	6	6	–	7
19	14	–	5	5	5	–	6	–	–	–	–	4
20	14	–	4	–	3	5	–	6	–	–	–	4
21	14	–	–	3	–	–	–	6	5	–	–	3
22	14	–	–	–	–	–	–	–	–	–	–	0
23	14	–	–	–	3	–	3	–	–	–	–	2
24	14	–	–	–	–	–	–	–	–	–	–	0
25	14	–	–	–	–	–	–	–	–	–	–	0
26	14	–	–	–	–	–	–	–	–	–	–	0
27	14	–	–	4	–	4	–	–	6	–		3
28	14	–	–	–	–	–	–	–	–	–		0
29	14	–	–	–	–	–	–	–	–	–		0
30	14	–	–	–	–	–	–	–	–	–		0
31	14	–	–	–	–	–	–	4	6	–		2
32	14	–	–	–	–	–	–	6	6	6		3
33	14	–	–	–	–	–	–	–	–	–		0

TMEV-infected brain samples were acquired from adult C57BL/6J mice. Seizure monitoring was initiated at 3 dpi and extended until the point of animal sacrifice or up to a maximum duration of 7 dpi.

### 2.3 Sample collection

Mice were transcardially perfused at various time points (3, 7, and 14 dpi), thus the maximum age difference between the groups was 11 days. Briefly, mice were deeply anesthetized via intraperitoneal pentobarbital injection or sacrificed through isoflurane inhalation. After cessation of breathing, transcardial perfusion was carried out using 1x phosphate-buffered saline (PBS; Thermo-Fischer), followed by a 4% paraformaldehyde [Sigma-Aldrich (PFA)] solution. Brains were then resected and stored overnight (ON) in 4% PFA at 4°C. On the subsequent day, brains were placed in vials containing 30% cold sucrose (Sigma-Aldrich) and stored at 4°C until complete dehydration. Hippocampi of both TMEV-infected and CTR mice were sectioned into 40 μm thick coronal slices using a freezing microtome (HM 400, MICROM GmbH, Germany). Slices were cut approximately from Bregma −1.22 to −3.80 mm (Paxinos and Franklin, 2004). These slices were divided into 8 series to represent the hippocampal dorso-ventral extent (a section every 280 μm per series). Every vial was pre-filled with cryoprotective solution [30% glycerol, 30% ethylene glycol, and 40% PBS (Roth, Germany)] and, after the procedure, contained the same number of slices. The vials were then stored at −20°C upon use. In this study a total of 33 TMEV injected mice divided across different dpi groups and 9 CTR mice were used.

### 2.4 Immunohistochemistry (IHC)

Theiler's Murine Encephalomyelitis Virus infected and CTR hippocampal slices were washed five times for 15 min with 1x PBS to remove the cryoprotective solution and residual PFA. After permeabilization and blocking (1 h, RT, constantly mixed) with 10% normal goat serum [Merck, Germany, Cat. #: S26-100ml, Lot #: 4064259 (NGS)] in 0.2% Triton X-100 [Merk, Germany, Cat. #: T8787-100ml, Lot #: SLCJ6163 (PBST)], samples were incubated ON at room temperature in 1x PBST containing 4% NGS and primary antibodies while being constantly mixed. Depending on the experimental design, different primary antibodies were used per one of the eight series of hippocampal sections: guinea pig anti-Doublecortin [Merck, Germany, Cat. #: AB2253, Lot #: 3850474, (DCX), neuronal progenitor cells, 1:1500], mouse anti-Nestin [Merck, Germany, Cat. #: MAB353, Lot #: 4040753, (NES), NSC 1:200] (Merck, Germany), rabbit anti-KI-67 (Abcam, Germany, Cat. #: Ab15580, Lot #: GR3397465-1, proliferative cells, 1:1000), and rabbit anti-Ionized Calcium-Binding Adapter Molecule 1 [Abcam, Germany, Cat. #: Ab178847, Lot #: GR3229566-16 (IBA1), microglia, 1:1000]. The following day, slices were washed three times for 15 min with 1x PBST and incubated for 2 h at RT in 1x PBS with 4% NGS and secondary antibodies conjugated with Alexa Fluor-488 (Invitrogen, Germany, Cat. #: A11008, Lot #: 2557379) and Alexa Fluor-568 (Invitrogen, Germany; Cat. #: A11011, Lot #: 2500544) dilution 1:1000 each, constantly mixed. Samples were then washed again three times for 15 min in 1x PBST and two times for 15 min in 1x PBS. Slices were mounted on slides and the cell *nuclei* were labeled using Fluoromount G^®^ containing DAPI (Invitrogen, Germany, Cat. #: 4959, Lot #: 142202). Finally, samples were covered with coverslips and stored at 4°C before the imaging process. For Glial Fibrillary Acidic Protein (GFAP, astrocytic marker) labeling, a Mix-n-Stain™ CF™647 kit (Sigma-Aldrich, Germany, Cat. #: MX647S20) was used. This disposable kit allowed for mixing the primary antibody (Mouse anti-GFAP, chosen dilution 1:2000) with the secondary (conjugated with Alexa Fluor-647) before use, thus avoiding the need for a second day of incubation. For each round of experiments, one slice was used as a negative control to evaluate the quality of the staining.

### 2.5 Imaging

Hippocampal slices processed as described above were used for imaging acquisition. Single-plane images of one series per animal (8 slices in total per animal) labeled for DCX, KI-67 and IBA1 were acquired by using a fluorescent microscope (Leica DMi8^®^, Leica, Germany). The hippocampal DG was visualized at 20x magnification with the focus corrected for all the channels. One image of all three channels was acquired [1,024 × 1,024 pixels (px), 1.3 μm/px]. Differently, the colocalization of GFAP^+^/NES^−^, NES^+^/GFAP^−^, and GFAP^+^/NES^+^ cells with KI-67 was acquired using a confocal laser scanning microscope (Leica SP8^®^, Leica, Germany). This approach was necessary as the signal from single-plane images acquired with the fluorescent microscope was insufficient to evaluate real colocalization. For acquisition, the laser's power was set to 1–2% and the photomultiplier tubes were adjusted to avoid saturation. The pinhole was set to 0.1 Airy Unit and the line average to 3. Then, a Z-stack of the entire DG (1,024 × 1,024 px, 0.75 μm/px, objective magnification: 20x, zoom factor: 0.75, distance in Z-direction: 2 μm) was acquired with a 20x objective. For this set of experiments, one dorsal and one ventral both ipsi- and contra-lateral hippocampal slice per animal per experimental group at the same coordinates were acquired as representative of the whole dorsal or ventral area. Regardless of the microscope used, all settings were kept constant throughout the acquisitions.

### 2.6 Imaging analysis

All acquired images were analyzed in a randomized order using ImageJ by an experimenter blinded to the animal ID and group allocation. Each sample acquisition was split into different channels with DAPI labeling used to identify the DG region. For single-plan acquisitions, a region of interest (ROI) was defined by tracing the external boundaries of the DG *stratum granulare* (SG) in each analyzed image and the corresponding area was calculated. For Z-stack acquisitions, the DG area was measured in the central imaged plane. Cell counts within the DG ROI were performed manually on single-plane acquisitions (DCX, KI-67, DCX/KI-67) and on different stacks (GFAP/KI-67, NES/KI-67, and GFAP/NES/KI-67), or on single-plane acquisitions (IBA1) using the “Analyse Particle” function. Additionally, the “Analyse Particle” was also used to calculate the area of each detected IBA1 cell. To this extent, the same ROI defined for calculating the DG area was used to identify the mean fluorescence intensity of the IBA1 labeling separately for each sample in the same experimental group. Then, the IBA1 channel was selected, filtered with the “Gamma filter” set to 1.5 and the “Gaussian Blur” set to 1 to improve image quality. Subsequently, twice the detected mean fluorescent intensity of the entire IBA1 dataset per group was used to threshold the images. To analyze both the number and area of IBA1 labeling, the “Analyse Particles” function was used, with the particle size, expressed as “pixel units,” set to 8 to remove background non-specific signals. This cutoff was chosen based on resolution limitation of the fluorescent microscope (Supplementary Figures 1E–G). Independent of the counting method, DCX, KI-67, and IBA1 cell density was calculated as a ratio between the number of positive cells per image and the ROI defined around the DG area of the same analyzed images. The result was then multiplied by a factor of 1,000,000 and the average per animal, expressed as cells/mm^2^, was calculated and used for statistical analysis. Differently, the density of colocalizing GFAP^+^/NES^−^, NES^+^/GFAP^−^, and GFAP^+^/NES^+^ cells with KI-67 was calculated within the Z-stack DG volume and approximated to mm^3^ by multiplying the resulting cell density of each analyzed image by a factor of 1,000,000,000. The approximated density was then averaged per animal, expressed as cells/mm^3^ and used for statistics.

To analyze the percentage of KI-67^+^ (proliferating) DCX^+^, GFAP^+^/NES^−^, NES^+^/GFAP^−^, and GFAP^+^/NES^+^ cells, a ratio of the total number of detected and manually counted double-positive cells for each specific marker to the total number of detected and manually counted KI-67^+^ cells in the DG for each imaged brain slice per animal was calculated. This ratio was then multiplied by 100 to obtain a percentage per imaged DG and subsequently averaged for each animal across the different groups. The final percentage per animal was used for statistics.

To evaluate possible aberrant neuronal progenitor cell migration, three reference lines were manually defined across the DG in each acquired image: one at the top, one at the center, and one at the bottom of the SG. Two additional lines were drawn between these, dividing the SG into 4 distinct layers (Figures 3A, B). Cells were assigned an arbitrary migration score (1 = normal migration, 4 = completely aberrant migration) based on their position within the defined layers. A score of 1 was assigned to cells located at the interface between the SG and the DG hilus. A score of 2 was given to cells situated between the first and the third lines, without touching the first line but crossing or touching the third. Cells between the third and the fifth lines received a score of 3, while a score of 4 was assigned to cells outside the SG, either in the molecular layer of the DG or within the hilus. Finally, the total number of scored cells per layer and hemisphere (ipsilateral or contralateral to the TMEV injection) was expressed as cell density. This was calculated as the number of manually counted cells per score per image (8 slices per animal across consistent series for all experimental groups), normalized to the DG area in the corresponding image. To standardize the data, the resulting value was multiplied by a factor of 1,000,000 to yield cell density in cells/mm^2^. For each animal, we determined the average density for each score, and group means were subsequently calculated. To express the results as percentages, the total number of cells across all four scores within each experimental group (3, 7, 14 dpi, and CTR), separated by hemisphere, was defined as 100%. The proportion of cells assigned to each score was then expressed as a percentage of this total, which was used for descriptive analysis.

### 2.7 Statistics

Data were unblinded and all statistical analyses were performed using GraphPad Prism Version 9. Normality distribution of the data was tested using the Shapiro-Wilk test. Multiple comparisons between normally or non-normally distributed data were further analyzed using either One-Way ANOVA followed by Bonferroni's multiple comparisons test or Kruskal-Wallis ANOVA followed by Dunn's multiple comparisons test. For comparisons between two groups, either an Unpaired *t*-test or Mann–Whitney test was used. Correlation analyses were conducted utilizing either the Pearson correlation coefficient or the Spearman correlation method, contingent upon the characteristics of the data distribution. Statistical significance was set at the adjusted (multiple comparisons) or exact *P*-value ≤ 0.05.

## 3 Results

### 3.1 Seizure occurrence after infection

The TMEV model was employed due to its translational relevance as a model of viral encephalitis-induced seizures. In this paradigm, intracerebral TMEV infection in C57BL/6J mice leads to the initiation of acute behavioral seizures in 50–70% of mice within a defined window spanning 3 to 8 dpi, followed by a seizure-free latency period. Around 14 dpi, the virus is cleared presumably due to activation of the adaptive immune response (DePaula-Silva et al., 2017). Acute seizures were recorded and are detailed in Table 1. To align with the study's core objective of examining the effects of virus infection and/or the occurrence of seizures on adult neurogenesis, each cohort included mice with and without seizures. Since seizure observation began at 3 dpi, animals from this group were monitored for seizures once before sacrifice and denoted as “+/–.”

### 3.2 Impact of TMEV infection on hilar cell proliferation

To comprehensively investigate the effect of TMEV infection on cell proliferation dynamics during the acute infection period (3–14 dpi), we conducted an analysis of KI-67^+^ cells in hippocampal slices from TMEV-infected mice at distinct time points: 3 dpi (*n* = 4), 7 dpi (*n* = 13), 14 dpi (*n* = 16; Table 1 and Figure 1). We included hippocampal slices from age-matched CTR mice (*n* = 5) to establish a baseline comparison for cellular proliferation. KI-67, a nuclear protein expressed throughout active phases of the cell cycle (G1, S, G2, and mitosis), remains undetectable in quiescent cells (G0; Bruno and Darzynkiewicz, 1992; Bullwinkel et al., 2006; Cuylen et al., 2016; Darzynkiewicz et al., 2015; Scholzen and Gerdes, 2000; Sobecki et al., 2016), making it a reliable marker for assessing cell proliferation. Cell density analyses were performed on the DG of all processed hippocampal samples (Figures 1F–I), stratified based on total average (Figure 1F), seizures detection (Figure 1G), dorsal/ventral (Figures 1H, I), and left/right hippocampus (data not shown). Notably, TMEV infection itself, rather than the occurrence of seizures, induced alterations in cell proliferation during the early post-virus infection stages, specifically at 14 dpi, when compared to the CTR group (Figures 1F, G). During the acute infection phase, cell proliferation in the dorsal DG was significantly reduced at 14 dpi in comparison to 3 dpi and to CTR (Figure 1H). No statistically significant differences in cell proliferation were observed between dorsal and ventral slices at any time point (data not shown). Additionally, no discernible variations in KI-67 expression were detected between the dorsal and ventral regions among both seizing and non-seizing mice (data not shown). An exploration into potential correlations between DG KI-67 cell density in seizing mice (including total, dorsal, or ventral) and seizure-related parameters such as number of seizures, seizure onset, and cumulative seizure score did not yield statistically significant findings (Supplementary Figures 2A, C, E).

### 3.3 TMEV infection immediately impairs DG neuronal progenitor density

**Figure 2:**
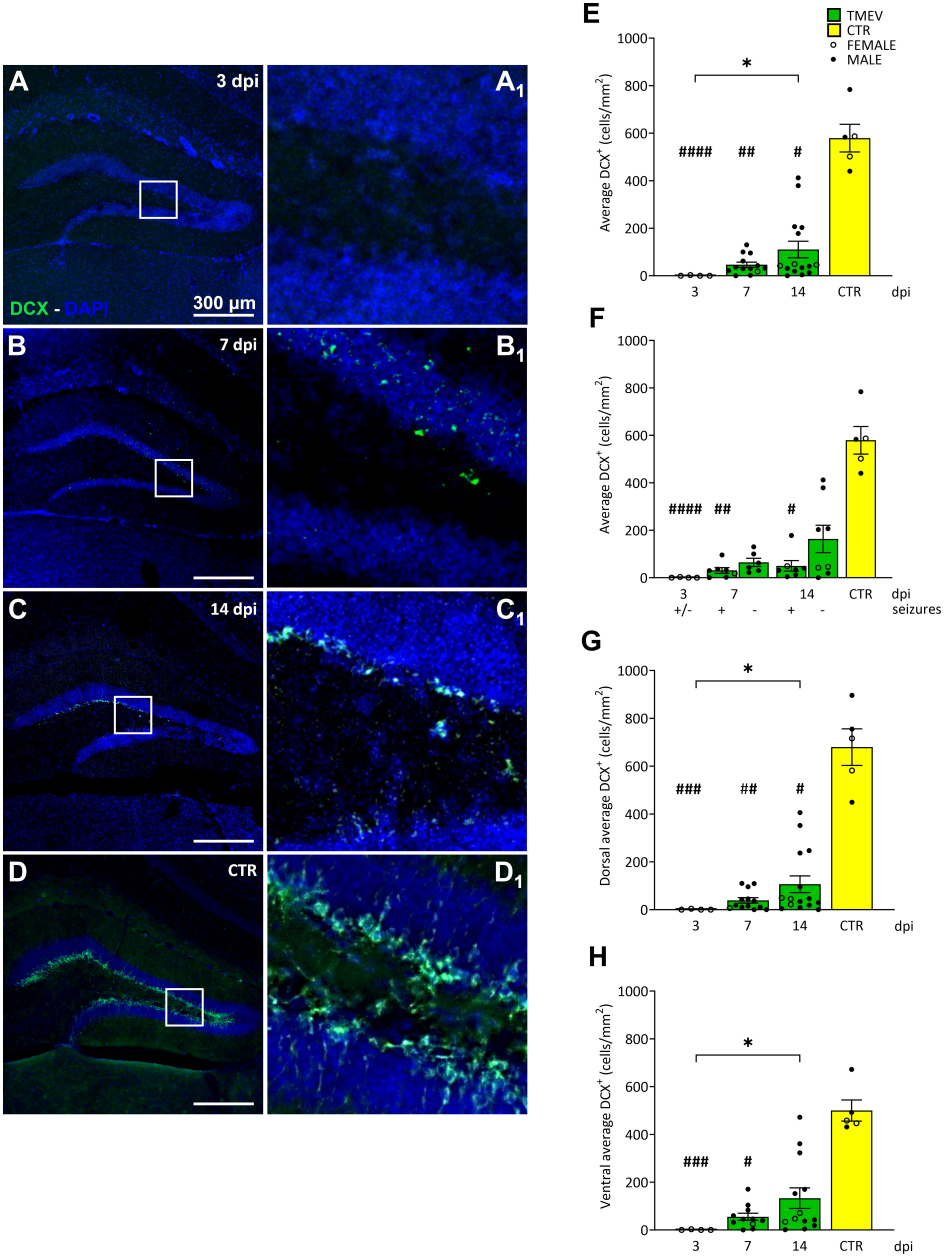
Theiler's Murine Encephalomyelitis Virus infection severely impacts neuronal progenitor proliferation. **(A–D)** Representative images showing IHC of DCX in C57BL/6J-mouse hippocampal slices at different time points post infection [3 dpi (*n* = 4) **(A)**, 7 dpi (*n* = 13) **(B)**, 14 dpi (*n* = 16) **(C)**, and CTR (*n* = 5) **(D)**]. **(E)** Average DCX^+^ cell density in TMEV-infected mice at different dpis. **(A**_**1**_**–D**_**1**_**)** Insets show magnified views of the corresponding ROIs in **(A–D)**. Immature neurons were significantly reduced over the acute phase of infection compared to CTR (3 dpi vs. CTR: ####*p* < 0.0001, 7 dpi vs. CTR: ##*p* = 0.0047, 14 dpi vs. CTR: #*p* = 0.0259). DCX cell population slowly increased over time (3 dpi vs. 14 dpi: *p = 0.0376). **(F)** Differences in DCX expression were mainly due to the occurrence of seizures [3 dpi (+/–) vs. CTR: ####*p* < 0.0001, 7 dpi (+) vs. CTR: ##*p* = 0.0027, 14 dpi (+) vs. CTR #*p* = 0.0223]. **(G, H)** Neuronal progenitor cell density analysis in both dorsal **(G)** and ventral **(H)** hippocampi. A partial recovery was observed over the different time points in both dorsal and ventral hippocampi [**(G)** 3 dpi vs. 14 dpi: *p = 0.0404; **(H)** 3 dpi vs. 14 dpi: *p = 0.0374]. The impact on neuronal progenitors seemed to be more severe in the dorsal hippocampal areas than in the ventral ones [**(G)** 3 dpi vs. CTR: ###*p* = 0.0001, 7 dpi vs. CTR: ##*p* = 0.0033, 14 dpi vs. CTR: #*p* = 0.0307; **(H)** 3 dpi vs. CTR: ###*p* = 0.0001, 7 dpi vs. CTR: #*p* = 0.0125]. The scale bar in **(A)** also applies to **(B–D)**. Filled circles indicate male mice, while hollow circles denote females. Data are mean ± SEM, normality was tested using the Shapiro-Wilk test, statistical analyses were performed using Kruskal-Wallis test followed by Dunn's multiple comparison test. Statistical significance was set at the adjusted *P*-value ≤ 0.05.

Next, we sought to elucidate the impact of TMEV infection on DG neuronal progenitors (DCX^+^; Figure 2). DCX is a microtubule-associated protein expressed by actively dividing neuronal precursor cells and immature neurons in both embryonic and adult brains, maintaining stable expression as the cells differentiate into neurons (Brown et al., 2003). Quantitative cell density analyses unveiled a striking and immediate depletion of DCX^+^ neuronal progenitor cells in the DG during the acute phase of TMEV infection compared to the CTR group (Figures 2E–H). At 3 dpi, the depletion was nearly complete, indicating a rapid and drastic effect on the neuronal progenitor population. However, by 7 and 14 dpi, a partial recovery was observed, with DCX^+^ cell density at 14 dpi being significantly higher than at 3 dpi across the total DG, as well as within the dorsal and ventral hippocampal regions (Figures 2E, G, H). Despite this recovery, the density of DCX^+^ cells at 14 dpi remained significantly lower than that in the CTR group, except in the ventral hippocampal area (Figures 2E, G, H). To further investigate the potential influence of seizure activity on neuronal progenitor cells in TMEV-infected brains, we compared mice that developed seizures with infected non-seizing mice (Figure 2F): Interestingly, mice experiencing seizures exhibited a more pronounced reduction in neuronal progenitor cell density compared to the CTR group. No significant differences were observed between the dorsal and ventral regions in seizing and non-seizing mice (data not shown). Consistent with our findings using KI-67 as a marker, correlation analyses between DG DCX cell density in seizing mice (total, dorsal, or ventral) and seizure parameters did not yield statistically significant results (Supplementary Figures 2B, D, F).

### 3.4 Neuronal progenitor cell migration is affected by TMEV infection

**Figure 3:**
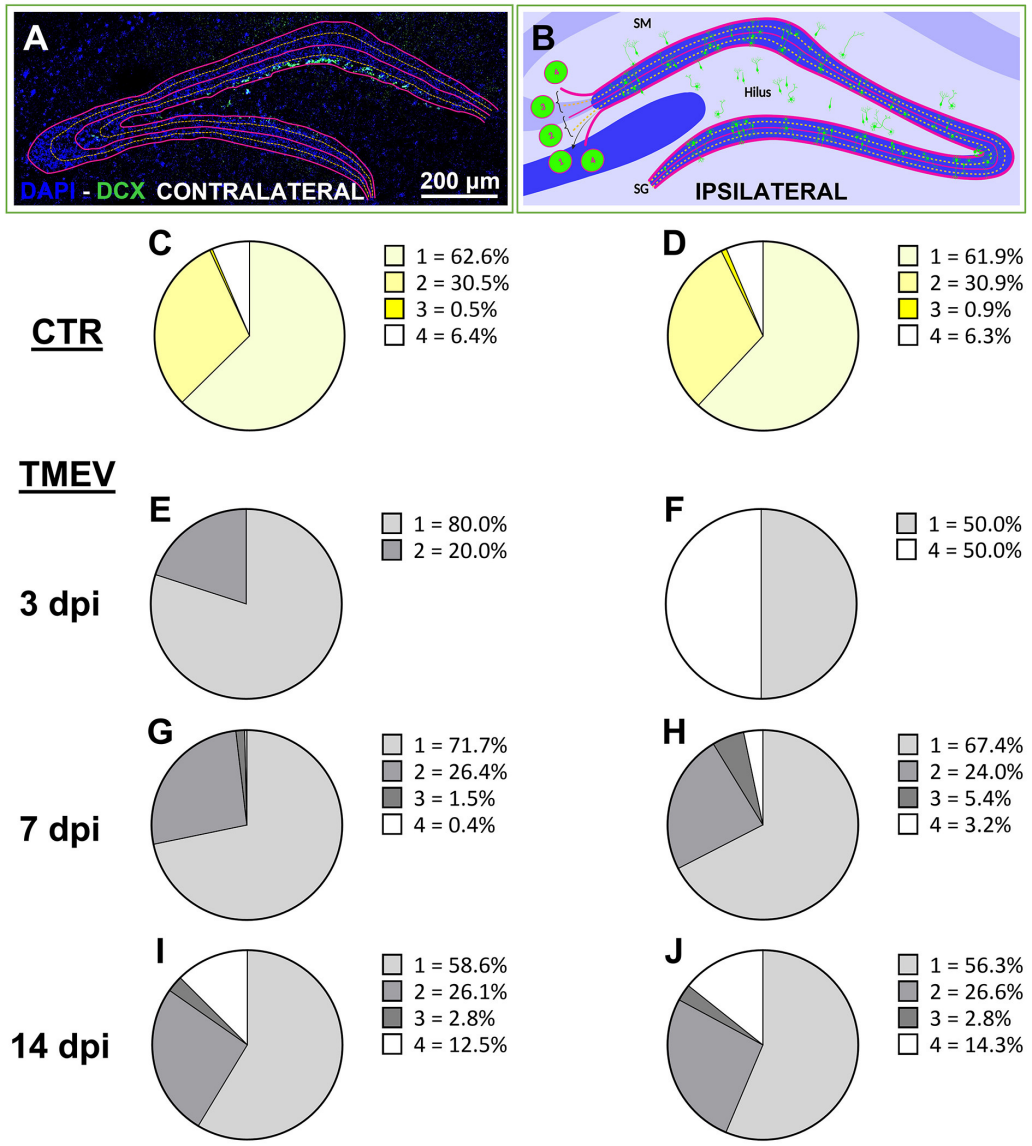
Neuronal progenitor cells aberrantly migrate over the acute phase after TMEV infection. **(A, B)** Aberrant migration was evaluated by analyzing ipsilateral and contralateral DG labeled for DCX at different time points [3 dpi (*n* = 4), 7 dpi (*n* = 13), 14 dpi (*n* = 16) compared to CTR (*n* = 5)]. **(C, D)** CTR showed identical score in both hemispheres. **(E, G, I)** In the acute phase of infection (from 3 to 14 dpi), the contralateral side showed a smoother transition of aberrant neuronal progenitor cell migration over the analyzed time points if compared to the ipsilateral side **(F, H, J)** which showed a completely aberrant migration already at 3 dpi **(F)**. **(I, J)** At 14 dpi, the contralateral and the ipsilateral side of infection displayed identical migration scores.

To evaluate the repercussions of TMEV infection on neuronal progenitor cell migration, we scrutinized the migration pattern of DCX^+^ cells on both the contralateral (left hemisphere) and ipsilateral (right hemisphere) sides relative to the TMEV injection (3 dpi (*n* = 4), 7 dpi (*n* = 13), 14 dpi (*n* = 16), as well as in CTR mice (*n* = 5) as shown in Figures 3A, B and expressed as percentage. Our analysis unveiled that CTR mice exhibited a parallel stratification of migrating neuronal progenitor cells in both analyzed hemispheres (Figures 3C, D). Indeed, over 90% of cells resided within regions categorized as score 1 or 2, characteristic of typical DCX^+^ cell locations, such as the subgranular zone of the DG and the ventral part of granule cell layer itself. In contrast, the migration pattern in infected animals was changed (Figures 3E–J), especially on the ipsilateral hemisphere to the infection: a migration score 4, representing neuronal progenitor migration outside of the typical areas within or below the granule cell layer (e.g., toward the hilus or *stratum moleculare*) was already visible at early time points (Figures 3F, H, J). At 3 dpi, despite very few DCX^+^ cells remaining after infection, a discernible aberration in the migratory behavior of DCX^+^ cells was evident (Figure 3F): 50% of the detectable DCX^+^ cells exhibited a migration score 4 in TMEV-infected mice. Neuronal progenitor cell migration on the contralateral side exhibited a smoother progression across various scores during the acute infection phase (Figures 3E, G, I). Specifically, migration score 4 was observed in the contralateral side starting at 14 dpi (Figure 3I). By this time point, the distribution of cells across different migration scores was almost equivalent in both sides (Figures 3I, J), with a notable surge in score 4 cells relative to prior time points. Analysis performed between seizing and non-seizing mice did not show any significance (data not shown).

### 3.5 TMEV infection impacts on the proliferative state of different classes of cells

**Figure 4:**
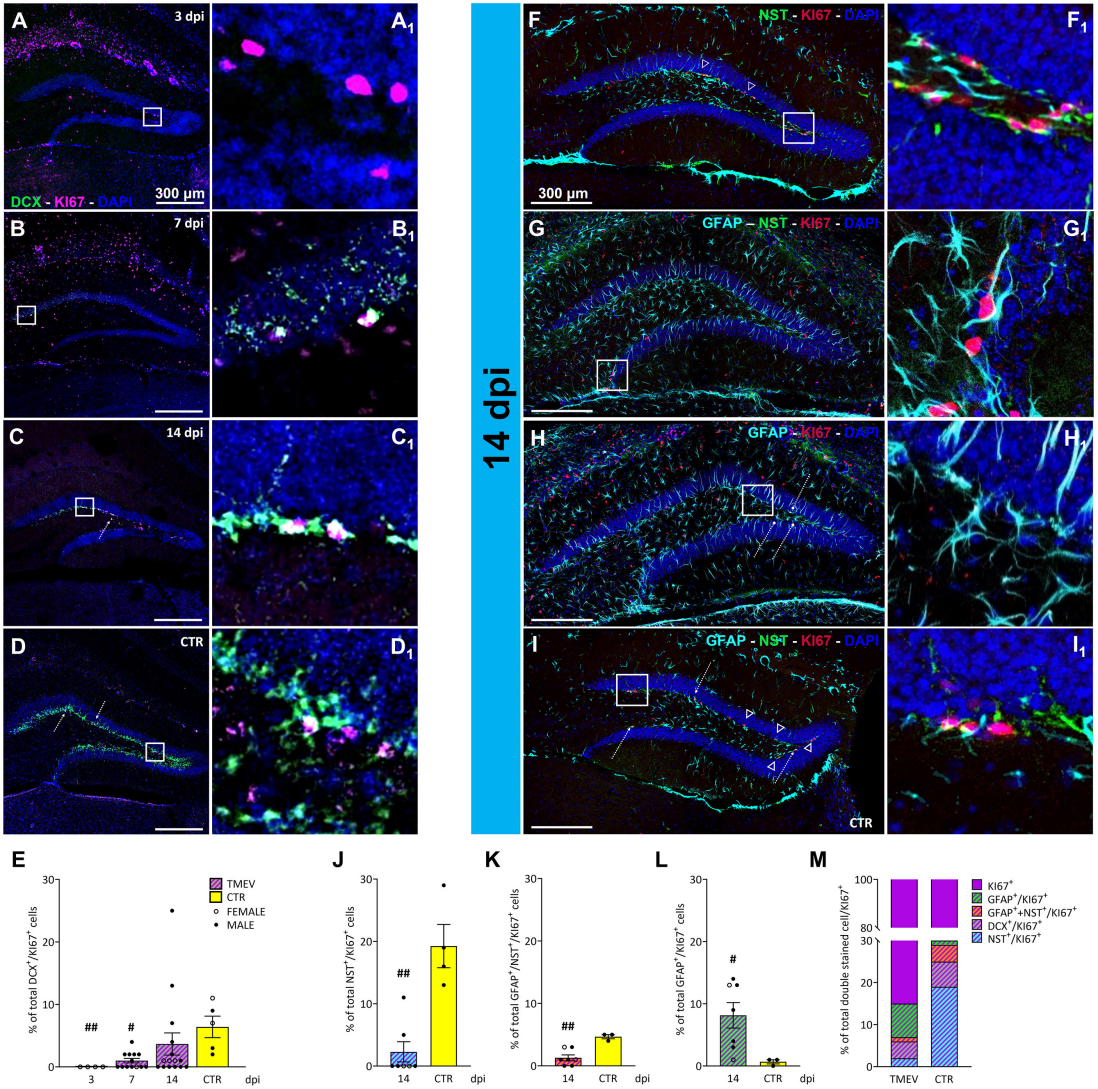
Proliferation state of different classes of cells during TMEV infection. **(A–D)** Representative images showing IHC of DCX and KI-67 in C57BL/6J-mouse hippocampal slices at different time points post infection [3 dpi (*n* = 4) **(A)**, 7 dpi (*n* = 13) **(B)**, 14 dpi (*n* = 16) **(C)**, and CTR (*n* = 5) **(D)**]. **(A**_**1**_**–D**_**1**_**)** Insets show magnified views of the corresponding ROIs in **(A–D)**. **(E)** Percentage of DCX^+^/KI-67^+^ analyzed in all the TMEV-infected mice divided per time points. Neuronal progenitor cells demonstrated significantly reduced proliferation at 3 and 7 dpi compared to CTR (3 dpi vs. CTR: ##*p* = 0.0037, 7 dpi vs. CTR: #*p* = 0.0201). **(F–I)** Identification of proliferative NSC cells and mature/immature astrocytes by IHC of NES^+^/KI-67^+^ (arrowheads; **F**, **I**), GFAP^+^/NES^+^/KI-67^+^ (**G**), and GFAP^+^/KI-67^+^ (bullet arrows; **H**, **I**), in a sub-cohort (*n* = 7) of 14 dpi C57BL/6J-mouse hippocampal slices. **(F**_**1**_**–I**_**1**_**)** Insets show magnified views of the corresponding ROIs in **(F–I)**. **(J)** The percentage of proliferating NSC populations was significantly reduced in TMEV-infected mice compared to CTR (14 dpi vs. CTR: ##*p* = 0.0030) as well as **(K)** the percentage of proliferating GFAP and NES co-labeled forms (14 dpi vs. CTR: ##*p* = 0.0083). **(L)** The percentage of proliferating mature astrocytes was significantly higher compared to CTR (14 dpi vs. CTR: #*p* = 0.0333). **(M)** Normalized overview of the analyzed proliferating cell populations at the end of the acute phase following TMEV infection compared to CTR. The percentage of proliferating NSC (NES^+^/KI-67^+^), immature astrocytes (GFAP^+^/NES^+^/KI-67^+^), and immature neurons (DCX^+^/KI-67^+^) is significantly lower in TMEV-infected mice compared to CTR, conversely, the percentage of proliferating mature astrocytes (GFAP^+^/KI-67^+^) exhibited a striking increase in TMEV-infected mice compared to CTR. The scale bar in **(A)** also applies to **(B–D)** whilst the scale bar in **(F)** also applies to **(G–I)**. Filled circles indicate male mice, while hollow circles denote females. Data are mean ± SEM, normality was tested using the Shapiro-Wilk test, statistical analyses were performed either using Kruskal-Wallis test followed by Dunn's multiple comparison test **(E)** or Mann-Whitney test **(J–L)**. Statistical significance was set at the adjusted (multiple comparisons) or exact *P*-value ≤ 0.05.

**Figure 5:**
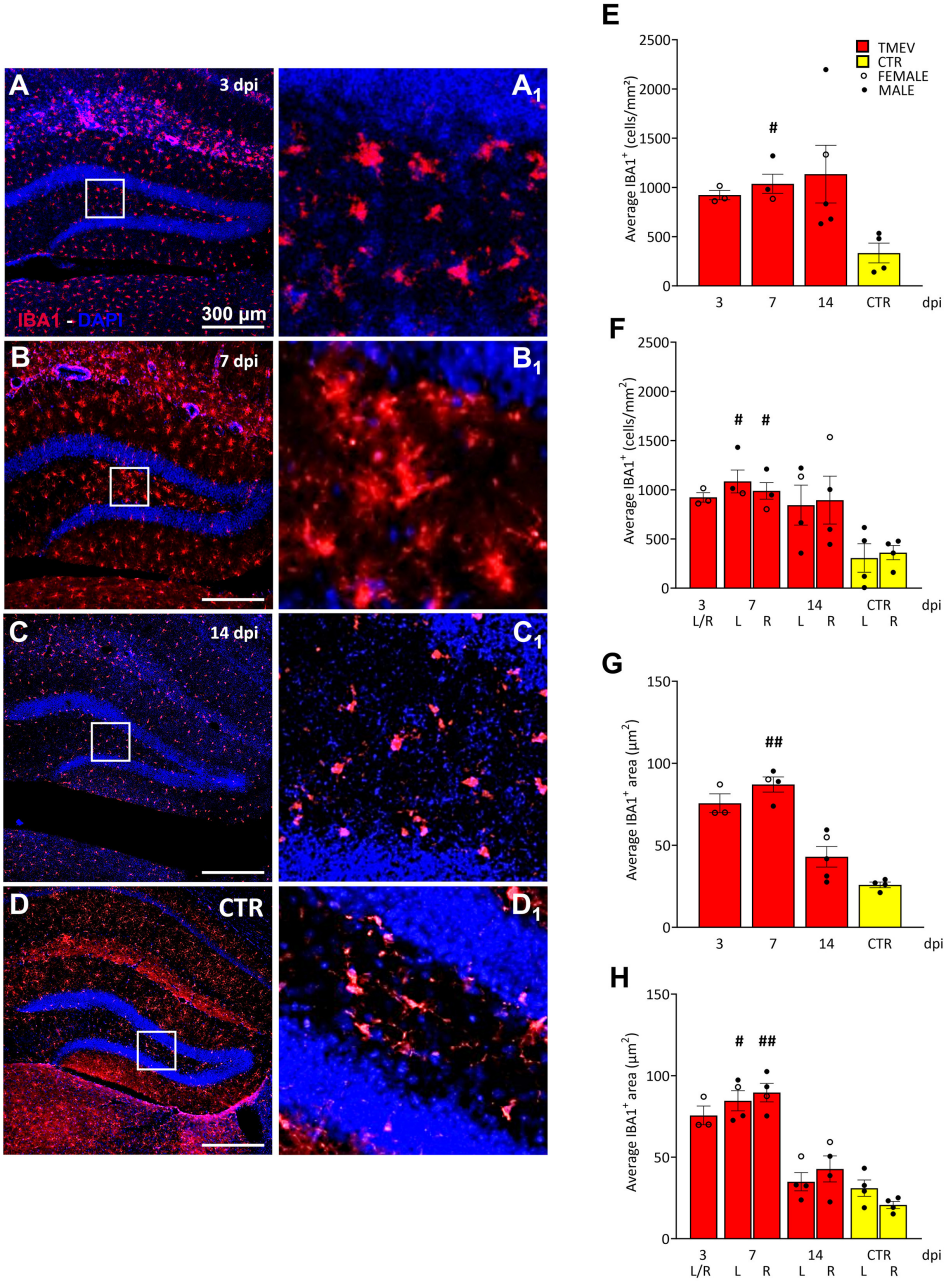
Theiler's Murine Encephalomyelitis Virus infection activates microglia response. **(A–D)** Representative images showing IHC of IBA1 in hippocampal slices of a subcohort of both seizing and non-seizing C57BL/6J-mice at different time points post infection [3 dpi (*n* = 3) **(A)**, 7 dpi (*n* = 4) **(B)**, 14 dpi (*n* = 5) **(C)**, and CTR (*n* = 3) **D**]. **(A**_**1**_–**D**_**1**_**)** Insets show magnified views of the corresponding ROIs in **(A–D)**. **(E)** DG IBA1^+^ cell density and **(G)** morphology showed an increase at 7 dpi relative to CTR [**(E)** 7 dpi vs. CTR: #*p* = 0.0451; **(G)** 7 dpi vs. CTR: ##*p* = 0.0032]. **(F, H)** This phenomenon occurred in both hemispheres **(F)** 7 dpi (L) vs. CTR (L) #*p* = 0.0372, 7 dpi (R) vs. CTR (R) #*p* = 0.0482; [**(H)** 7 dpi (L) vs. CTR (L) #*p* = 0.0445, 7 dpi (R) vs. CTR (R) ##*p* = 0.0017]. No significant differences between left (L) and right (R) as well as between cohorts were detected at any analyzed time point **(E–H)**. The scale bar in **(A)** also applies to **(B–D)**. Filled circles indicate male mice, while hollow circles denote females. Data are mean ± SEM, normality was tested using the Shapiro-Wilk test, statistical analyses were performed using Kruskal-Wallis test followed by Dunn's multiple comparison test. Statistical significance was set at the adjusted *P*-value ≤ 0.05.

After having found that immature neurons were depleted, we wanted to investigate whether they were replenished by cell proliferation. To investigate the impact of TMEV infection on neuronal progenitor proliferation, we conducted a focused analysis on the percentage of double-stained DCX^+^/KI-67^+^ cells. For this investigation, we utilized TMEV-infected mice from various time points (3 dpi (*n* = 4), 7 dpi (*n* = 13), 14 dpi (*n* = 16), compared to CTR (*n* = 5; Figures 4A–D). Quantitative analysis of the double-stained cells (Figure 4E) revealed that at 3 and 7 dpi, DCX^+^ neuronal progenitor cells demonstrated significantly reduced proliferation compared to CTR. This was accompanied by a progressive increase in proliferating neuronal precursors across the different time points, consistent with the upregulation of DCX expression (Figure 2). To further elucidate this phenomenon, we conducted region-specific analyses on the dorsal, ventral, left, and right DG regions (Supplementary Figure 4). The significant reduction in the percentage of proliferating neuronal progenitors observed at 3 and 7 dpi, compared to CTR, was consistent across all regions (Supplementary Figures 4A–D). Overall, only a small percentage of proliferative cells represented neuronal progenitors, even in the CTR group (6.4% of KI-67^+^ cells). This prompted us to assess the identity of the other proliferating cells, such as NSC (NES^+^/GFAP^−^), radial glia (GFAP^+^/NES^+^) and astrocyte-like cells (GFAP^+^/NES^−^), in order to determine the specific proliferating cell types. The rationale for selecting these cell types stemmed from literature suggesting that the stem cell niche is giving rise to either neuronal progenitor cells (in a physiological environment) or astrocytes (in a pathological environment; Hattiangady and Shetty, 2010). To gain deeper insights, we focused our analyses on 14 dpi for multiple reasons: (1) 14 dpi marks the endpoint for virus clearance in this model and represents the conclusion of the acute infection phase, (2) 14 dpi exhibited the highest expression of DCX^+^ cells during the acute phase, and (3) the 14-dpi cohort comprised both seizing and non-seizing mice. To conduct this experiment, we randomly selected 3–7 mice from each group (seizing and non-seizing), performed IHC on NES, GFAP, and KI-67 (Figures 4F–I), and analyzed the GFAP^+^/NES^+^/KI-67^+^, and GFAP^+^/NES^−^/KI-67^+^ cell populations, similar to our previous analysis of DCX^+^/KI-67^+^. Subsequent cell count analysis revealed that TMEV infection significantly reduced the percentage of proliferating NSC cell populations compared to CTR (Figure 4J) in all analyzed regions (Supplementary Figures 4E–H). Analysis of GFAP and NES co-labeled cells showed a significant reduction compared to CTR (Figure 4K), predominantly observed in the left hemisphere (Supplementary Figure 4K). Additionally, the percentage of proliferating astrocytes (Figure 4L) was significantly higher than in CTR, validating the presence of an inflammatory substrate, as indicated by our analysis of IBA1^+^ cell density and morphology (Figure 5). However, this increase in proliferating astrocytes was only statistically significant vs. CTR in the dorsal and ventral areas (Supplementary Figures 4M, N), while there was no difference between the infected right and left hemispheres (Supplementary Figures 4O, P). Despite conducting an analysis of the percentage of proliferating neuronal precursor cells, proliferating NSCs or proliferating astrocytes in relation to seizure-related parameters, such as number of seizures, seizure onset and cumulative seizure score, no statistically significant results were obtained (data not shown). To present a comprehensive overview of proliferating cells at the end of the acute phase following TMEV infection compared to CTR, we normalized the dataset for each individual cell marker used to detect proliferating cells to the KI-67^+^ total percentage (Figure 4M). This analysis yielded intriguing differences between CTR and TMEV-infected mice at 14 dpi. Specifically, not only did the percentage of proliferating early progenitors (NES^+^/KI-67^+^ and NES^+^/GFAP^+^/KI-67^+^) significantly decrease in TMEV-infected mice compared to CTR, but also the percentages of proliferating neuronal progenitor cells (DCX^+^/KI-67^+^) were notably lower. Conversely, the percentage of proliferating astrocytes (NES^−^/GFAP^+^/KI-67^+^) exhibited a striking increase in TMEV-infected mice compared to CTR.

### 3.6 Microglia activation in the mouse DG in the acute phase of virus infection

Since seizures did not seem to have a major effect on cell proliferation, contrarily to what was previously published in etiologically different models of seizures and epilepsy (Bengzon et al., 1997; Gray and Sundstrom, 1998; Parent et al., 2006; Parent and Murphy, 2008; Parent et al., 2002, 1997; Scott et al., 1998), we compared the rate of inflammation after virus infection to identify a potential influence on cell proliferation, and/or seizure occurrence. To achieve this, we examined a randomized sub-cohort of mice, consisting of both seizing and non-seizing animals (*n* = 3–5), over the course of infection from 3 to 14 dpi. CTR mice (*n* = 4) were included as a reference group for comparative analyses. As microglia play a crucial role in neuroinflammation (Bröer and Pauletti, 2024), we employed IBA1 as a well-established marker to assess reactive microglia (Figure 5). IBA1 is known to be upregulated during macrophage/microglia activation in various brain diseases (Hoogland et al., 2015; Ito et al., 1998; Lier et al., 2021), including TMEV infection (DePaula-Silva et al., 2021, 2019; Jafari et al., 2012; Loewen et al., 2016). In order to assess the extent of the inflammatory response occurring within the hippocampus during the acute stage of the infection (3–14 dpi), our investigation centered upon the quantification of IBA1 cell density and the characterization of alterations in their morphology (Supplementary Figures 1E–G). This approach was adopted with the dual purpose of identifying any potential augmentation in the presence of infiltrated microglial cells within the DG, while concurrently estimating their level of activation. This rationale is grounded in established literature, which stipulates that microglial activation engenders discernible shifts in cellular dimensions (as evidenced by an increase in cell size). By analyzing both the cell density and the area of each positive cell, we confirmed earlier work (Bröer et al., 2017, 2016; Cusick et al., 2017; DePaula-Silva et al., 2021; Hanak et al., 2019; Howe et al., 2022; Kirkman et al., 2010; Loewen et al., 2016; Sanchez et al., 2019; Waltl et al., 2018a,b) that a significant increase in recruited and activated immune cells was evident when comparing TMEV-infected mice with CTR at 7 dpi (Figures 5E–H). This increase in IBA1^+^ cell density and morphology was observed not only in the ipsilateral hemisphere, but also in the contralateral hemisphere to the site of virus injection (Figures 5F, H), indicating a bilateral microglial response during TMEV infection. We further sought to elucidate whether the microglial activation differed across distinct time points during the acute phase of infection: No statistical differences in IBA1 cell density and morphology were observed between 3, 7, and 14 dpi (Figures 5E–H), suggesting that microglia activation reaches a relatively stable state during this early phase of infection. Furthermore, we explored potential correlations between DG IBA1 cell density in seizing mice (total, dorsal, or ventral) and seizure-related parameters, such as number of seizures, seizure onset, and cumulative seizure score. However, no significant correlations were found in our analyses (data not shown). Moreover, the correlation analyses conducted between total DCX cell density and total IBA1 cell density (Supplementary Figures 3A, B) or IBA1 morphology (Supplementary Figures 3C, D) at 7 and 14 dpi did not attain statistical significance notwithstanding an apparent negative correlation trend evident in all depicted data.

## 4 Discussion

The primary objective of this investigation was to conduct an initial assessment of the impact of viral encephalitis-induced neuroinflammation and seizures on adult neurogenesis during the acute phase of infection. To achieve this objective, we employed a viral encephalitis model using male and female C57BL/6J mice which were subjected to intracortical injection of the neurovirulent DA strain of TMEV. This strain is known for its infection of CA1 and CA2 pyramidal neurons, resulting in substantial neuronal loss within the hippocampal region (Bröer et al., 2017, 2016; Gerhauser et al., 2019) and acute polioencephalitis (Dal Canto and Lipton, 1982; Jafari et al., 2012; Lipton et al., 2005). This viral spread coincides with significant microglial activation, macrophage infiltration, and the release of pro-inflammatory cytokines, which play a crucial role in exacerbating neuroinflammation and modulating neuronal excitatory patterns, thus contributing to the onset of acute seizures (Bröer et al., 2017, 2016; Cusick et al., 2017; DePaula-Silva et al., 2021, 2018; Di Nunzio et al., 2021; Hanak et al., 2019; Howe et al., 2022; Kirkman et al., 2010; Loewen et al., 2016; Sanchez et al., 2019; Waltl et al., 2018a,b). Following infection, ~50%−70% of C57BL/6J mice exhibit transient early (acute) afebrile seizures along with impaired motor function and coordination within 3–7 dpi. This is followed by a seizure-free period, viral clearance by the immune system within 2–3 weeks, and a subsequent reduction in seizure thresholds months after infection, as well as chronic epilepsy in about 25%−40% of infected mice (Batot et al., 2022). This model effectively recapitulates various early and chronic symptoms of virus-induced epilepsy observed in humans (Batot et al., 2022; Bröer et al., 2016; Gerhauser et al., 2019; Libbey et al., 2008; Stewart et al., 2012, 2010), and it is not subjected to sex-related differences (Bröer et al., 2016; Libbey et al., 2008), enabling us to investigate adult neurogenesis responses to viral infection in both seizing and non-seizing male and female animals.

To comprehensively dissect the phenomena occurring following viral infection, we conducted analyses in the DG at three distinct time points during the acute infection phase (3 dpi seizure onset, 7 dpi seizure peak, and 14 dpi virus clearance and several days after seizures end) to assess early neuroinflammation and/or seizure-induced neurogenesis modification. Notably, acute infection with the DA strain of TMEV *per-se* does not produce detectable effects on the DG, as assessed by magnetic resonance imaging and immunohistochemical analysis of viral antigen (Buenz et al., 2009), since the virus disseminates across the cortex, thalamus, and brainstem, as well as within the CA1-2 regions, and subiculum of the hippocampus (Batot et al., 2022; Buenz et al., 2009). Finally, although we acknowledge the limitations inherent in utilizing CTR mice and DMEM injected vehicle controls sourced from a different laboratory than the one which generated the TMEV model, it is crucial to highlight that the CTRs and DMEM injected mice employed in this study were age-matched, derived from the same genetic strain, and once compared to each other showed no statistical differences thereby ensuring the validity of our comparative analyses.

Our findings reveal several novel insights into the impact of viral encephalitis on adult neurogenesis: Employing KI-67, a reliable marker for proliferating cells expressed during mitosis (Bruno and Darzynkiewicz, 1992) our investigation showed a reduction in hilar cell proliferation during the acute phase of infection, aligning with earlier findings in different *in vitro* and *in vivo* models of CNS viral infections (Li et al., 2016; McGrath et al., 2017; Souza et al., 2016). Importantly, there were no significant disparities in KI-67^+^ density between mice experiencing seizures and those that did not, implying that, in this model, the infection itself or the inflammatory response to the viral infection might exert a more substantial influence on cell proliferation patterns during the acute phase of TMEV infection, as opposed to the occurrence of seizures. Indeed, data derived from various experimental models of epilepsy have consistently indicated an extensive acute increase in cell proliferation following prolonged seizures in rodent models as a possible compensatory response of the brain to mitigate the significant neuronal cell loss (Bengzon et al., 1997; Gray and Sundstrom, 1998; Parent et al., 2006; Parent and Murphy, 2008; Parent et al., 2002, 1997; Scharfman and Gray, 2006; Scott et al., 1998). TMEV infection does not produce status epilepticus, but rather short acute seizures, thus the divergence in seizure manifestation across models may account for the detected lower levels of cell proliferation, suggesting that increased cell proliferation in other epilepsy models may result from a synergistic effect of inflammation and prolonged or more severe seizures. In fact, while we cannot entirely exclude the possibility that the general inflammatory cascade in the TMEV model, once initiated and in presence of seizures, may differ from that observed in other epilepsy models (such as those induced chemically or electrically). It is important to acknowledge that the initial insult and mechanisms leading to inflammation and subsequent cell loss are inherently different: Both TMEV-induced and seizure-induced inflammation involve the activation of glial cells and the release of pro-inflammatory cytokines. However, TMEV-induced inflammation is driven by viral infection and the resulting immune response, with the virus infecting specific cell populations and leading to direct viral cytotoxicity and neuronal loss. The immune response involves microglial activation and peripheral immune cell infiltration, resulting in the production of pro-inflammatory cytokines and sustaining inflammation as the immune system attempts to clear the infection, which is achieved by the latest time-point of our study at 14 dpi (Bröer et al., 2017; Gerhauser et al., 2019; Vezzani et al., 2016). This phenomenon could likely induce cell death in other vulnerable neuronal populations such as newborn neurons. In contrast, in a non-infectious setting seizure-induced inflammation primarily arises from excessive neuronal activity leading to excitotoxicity.

Moreover, prior studies on various epilepsy animal models and humans also indicated a significant increase in neuronal progenitor cells during status epilepticus or acute seizures (Bengzon et al., 1997; D'Alessio et al., 2010; Gray and Sundstrom, 1998; Liu et al., 2018; Parent et al., 2006; Parent and Murphy, 2008; Parent et al., 2002, 1997; Scott et al., 1998), while our findings diverged: We observed a rapid depletion of neuronal progenitor cells within the DG during the acute phase of TMEV infection compared to the CTR group by using DCX, a microtubule-associated protein expressed in actively dividing neuronal precursor cells and immature neurons (Brown et al., 2003). At 3 dpi, this depletion was nearly complete, indicating a swift and significant impact on the neuronal progenitor cell population. Subsequent time points (7 and 14 dpi) showed a partial recovery of neuronal progenitor cells, but their density remained significantly lower than that observed in CTR mice. Interestingly, this phenomenon was more pronounced in the dorsal DG compared to the ventral region during the acute phase of infection, which is closer to the site of infection. In a comprehensive examination of the neurogenesis time course in C57BL/6J mice, a longitudinal study spanning 9 months revealed a consistent reduction in DCX expression on a monthly basis, while the KI-67/DCX ratio was constant throughout the entire investigative period (Ben Abdallah et al., 2010). Differently, in our model, we did see an increase in both DCX expression and the DCX^+^/KI-67^+^ cell ratio, prompting the assertion that the observed effects are a compensatory mechanism after initial depletion.

Remarkably, correlation analyses failed to yield statistically significant results between neurogenesis and the analyzed seizures parameters or inflammation across all examined time points. However, a notable decrease in DCX^+^ cell density was predominantly observed in seizing mice. Given the well-established detrimental interplay between inflammation and seizures in epilepsy (Löscher and Brandt, 2010; Vezzani, 2020), it is plausible that the reduction in DCX^+^ cell density in seizing mice reflects a combined seizures-inflammation phenotype rather than being attributable to either condition alone or to specific seizures related parameters. Our data align with the scientific literature on several viruses infecting the CNS: Sharma et al. (2002), Li Puma et al. (2019), and Garber et al. (2018) showed that, by infecting rodents, respectively, with Lymphocytic choriomeningitis virus, HSV-1 or WNV, neurogenesis was impaired long after the initial insult without seizure activity. Recent investigations conducted by Fernández-Castañeda et al. (2022) and Soung et al. (2022), utilizing murine and hamster models, respectively, to simulate SARS-CoV-2 CNS infections, revealed a noteworthy reduction in DCX^+^ cells within the SVZ of the DG as early as 5–7 dpi. Notably, like in our study, this effect manifested more prominently in the dorsal as opposed to the ventral region. This decline in DCX^+^ cells persisted up to 7 weeks post-infection. It is noteworthy that both in the hamster model of viral CNS infection and in the model used in our research, a significantly lower amount of DCX^+^/KI-67^+^ signal was recorded in the acute phase, while the hamsters did not show any seizures after infection, further substantiating the important role of inflammation on neurogenesis in the TMEV model.

Furthermore, multiple studies investigating the impact of SARS-CoV-2 on neurogenesis have elucidated that the deleterious consequences on hippocampal adult neurogenesis stem from the presence of reactive microglia/macrophages and an elevated concentration of interleukin-6 (IL-6). Empirically, IL-6 has been demonstrated to induce apoptosis in healthy adult hippocampal NSCs (Nikolopoulos et al., 2023), alongside the involvement of C-C motif chemokine ligand 11 (CCL11; Fernández-Castañeda et al., 2022; Monje et al., 2003; Villeda et al., 2011). Notably, Parajuli et al. (2015), postulated that CCL11 fosters microglial migration, initiating the production of reactive oxygen species and consequently precipitating neuronal demise. Given the lack of correlation between seizures and cell proliferation, we explored inflammation levels following virus infection as a potential influencing factor by evaluating the expression of the microglial marker IBA1 during the acute phase of infection. It is well-documented in the scientific literature that IBA1 expression undergoes upregulation during the activation of macrophages and microglia in the context of human epilepsy (Somani et al., 2021; van Vliet et al., 2018; Vezzani et al., 2011; Vezzani and Granata, 2005; Wirenfeldt et al., 2009), as well as in various epilepsy models induced either chemically, electrically or by viral infections (DePaula-Silva et al., 2021, 2019; Eyo et al., 2017; Hoogland et al., 2015; Howe et al., 2022; Ito et al., 1998; Jafari et al., 2012; Lier et al., 2021; Loewen et al., 2016; Vezzani et al., 2011; Vezzani and Granata, 2005; Wu et al., 2023). Consonant with the extant body of scientific knowledge, our findings reveal an augmentation in the population of IBA1^+^ cells and an elevated state of their activation during the acute phase of infection as displayed by our morphometric analysis (7 dpi). Due to the limited number of mice used in this analysis, we were unable to further differentiate between seizing and non-seizing mice. Future studies will include larger cohorts to better investigate differences in microglia activation in animals experiencing seizures vs. those that do not. While microglia activation, as an indicator of inflammation, is not inherently pro- or anti-neurogenic, the ultimate outcome depends on the balance between secreted molecules with pro- and anti-inflammatory actions (Ekdahl et al., 2009). During viral infections and in epilepsy, proinflammatory molecules such as interleukin-1β and the high mobility group box 1 are known to be released (Hernangómez et al., 2014; Kim, 2023; Pauletti et al., 2019; Vezzani et al., 2011; Vezzani and Granata, 2005) and to instigate a convergent signaling cascade which culminates in the activation of downstream pathways that intersect with Tumor Necrosis Factor pathways at the transcription factor nuclear factor κB (Gilmore, 2006; Vezzani et al., 2011) exerting influence over the expression of genes involved in cell viability, synaptic molecular reorganization, plasticity, and neurogenesis (O'Neill and Kaltschmidt, 1997). It is then plausible that proinflammatory molecules released during TMEV infection, took part in decreasing cell proliferation and neuronal progenitor cell population.

Furthermore, we assessed the migration of the remaining neuronal progenitors within the DG. Neurons born during and after seizures often display morphological and functional alterations, leading to long-lasting structural changes in hippocampal morphology and increased hyperexcitability, elevating the risk of subsequent seizures (Jessberger et al., 2007). One of them is the different migration behavior of the neuronal progenitor cells, which leads to ectopic proliferation (Jessberger et al., 2005), and subsequently to abnormal bursting and self-recurrent seizures (Bielefeld et al., 2019). Our analyses confirmed previous findings, as aberrant migration was already evident at 3 dpi in the ipsilateral side to the virus injection. Since migration analysis between seizing and non-seizing animals in the acute phase of infection did not show any significant differences (data not shown), it is likely that the results observed are driven by neuroinflammation. This hypothesis could explain why the migration score in infected mice diverges from the CTR within the acute phase of infection, which represents the time the immune system needs to clear the virus from the CNS (Batot et al., 2022; Bröer et al., 2016; Gerhauser et al., 2019; Libbey et al., 2008; Stewart et al., 2012, 2010). Thus, it would be important to include larger groups of mice to characterize the long-term effects of virus infection and subsequent epilepsy development on neurogenesis. EEG recordings, together with more specific analyses of inflammation markers over the time course of disease, will be essential to better understand the contributions of these factors.

Other crucial mechanisms contributing to a pro-inflammatory and a pro-epileptogenic microenvironment are the larger number of NSCs that, during seizure occurrence, accelerate their division and symmetrically produce more astrocytes than neuronal progenitor cells, leading to the depletion of the stem cell niche (Amiri et al., 2012; Hattiangady et al., 2004; Hattiangady and Shetty, 2010; Loewen et al., 2016; Overstreet-Wadiche et al., 2006; Sierra et al., 2015). NSCs display remarkable heterogeneity in their proliferative state, ranging from self-renewal asymmetric divisions by generating progenitor cells committed to neuronal differentiation, to symmetric divisions giving birth to astrocytic progeny (Bao and Song, 2018; Encinas et al., 2011). Since our results showed a very low percentage of proliferating neuronal progenitors (DCX^+^/KI-67^+^), we analyzed the proliferative NSC pool. Analysis of NSCs (NES^+^, GFAP^+^/NES^+^) at 14 dpi revealed a significant reduction in proliferation, accompanied by a significant increase in proliferating astrocyte-like cells (GFAP^+^/NES^−^) compared to CTR. These findings are intriguing, as the period of virus clearance coincides with heightened inflammation during the acute phase of infection, as also supported by our data on IBA1^+^ cell density and activation. Given the lack of direct correlation between the number of IBA1^+^, DCX^+^, NES^+^, GFAP^+^/NES^+^, and GFAP^+^/NES^−^ cells in the DG and seizure parameters during the acute phase of TMEV infection, it is conceivable that viral insult, rather than seizure occurrence, primarily triggered the neuroinflammatory response committing NSCs toward the symmetric division and, subsequently, affecting neuronal progenitor cell population. In fact, numerous studies on different viral infections have shown IL-1α and IL-1β to induce NSCs to favor the astrocytic lineage rather than the neuronal lineage both *in vitro* and *in vivo* either via downstream induction of the transcription factor (Garber et al., 2018; Green et al., 2012; Peng et al., 2011) and, at the same time, impacting on neurogenesis (Garber et al., 2018; Wang et al., 2007; Wu et al., 2012). Furthermore, it is noteworthy to mention that astrocytes are not the only proliferating cells during TMEV infection. Recent findings by Bell et al. (2020) have demonstrated that TMEV-induced infection leads to an augmented proliferation of NG2 glia, precursors of oligodendrocytes. However, it is recognized that depending on the developmental stage and the specific brain region, NG2-glia can also give rise to astrocytes (Zhu et al., 2008, 2011) or persist as self-renewing NG2-glia (Simon et al., 2011).

## 5 Conclusion

To date, the multifaceted interactions of viral CNS infection, as well as its accompanying neuroinflammation, and subsequent acute and chronic seizures and their impact on neurogenesis have not been thoroughly explored, making this study a pioneering contribution to research on one of the most common causes of seizures and epilepsy. Our study reveals a complex interplay between TMEV infection, inflammation, seizures, and the dynamics of adult neurogenesis within the DG. Notably, our findings, for the first time, unveiled a distinctive temporal dimension in the regulation of cell proliferation. Specifically, we observed that the overall cell proliferation between TMEV-infected mice and their uninfected CTR significantly decreased during the acute phase following infection. Subsequently, a divergence in the fate of proliferating cells became evident between TMEV-infected and CTR cohorts:

- The population of neuronal progenitors was almost entirely absent at 3 dpi, but started to replenish over time. However, the density of DCX^+^ cells did not reach CTR levels. Further exploration of proliferating neuronal progenitors, identified by the co-expression of DCX and KI-67 (DCX^+^/KI-67^+^), disclosed that only a minor fraction of KI-67^+^ cells co-localized with DCX in both TMEV-infected and CTR mice. Additionally, remaining DCX^+^ cells exhibited aberrant migratory patterns as early as 14 dpi.
- The population of proliferating NSCs was significantly reduced in the DG of TMEV-infected mice at 14 dpi in comparison to CTR mice.
- The number of proliferating astrocytes was notably elevated in the TMEV group.

Furthermore, a conspicuous increase in activated microglia was evident as early as 7 dpi compared to CTR. Our results suggest that, in this animal model, the viral infection initially, followed by the occurrence of seizures, drives the observed phenotypic changes. However, we acknowledge the necessity of rigorous control over seizure frequency via EEG monitoring to better determine seizures impact on adult neurogenesis after TMEV infection. Additionally, a comprehensive study of the chronic phase of the disease is essential. Since not all infected animals develop chronic seizures, analyzing the long-term effects of the initial viral infection on the hippocampal cytoarchitecture is crucial for identifying potential therapeutic approaches.

## Data Availability

The raw data supporting the conclusions of this article will be made available by the authors, without undue reservation.

## Supplementary material

Supplementary Figure 1 Comparison between naïve and vehicle-injected mice and example of “Analyse Particle” analysis. **(A–D)** Comparative analyses between the naïve (CTR) and vehicle-injected experimental groups were performed to confirm that injection procedure itself did not influenced the analyzed parameters. No statistically significant differences were observed between naïve and vehicle-injected animals in: **(A)** Average KI-67^+^ cell density [NAÏVE (*n* = 5) vs. VEHICLE (*n* = 3) *p* = 0.4968], **(B)** Average DCX^+^ cell density [NAÏVE (*n* = 5) vs. VEHICLE (*n* = 6) *p* = 0.1172], **(C)** Average IBA1^+^ cell density [NAÏVE (*n* = 4) vs. VEHICLE (*n* = 4) *p* = 0.8857], and **(D)** Average IBA1^+^ Area [NAÏVE (*n* = 4) vs. VEHICLE (*n* = 4) *p* = 0.4434]. Data are mean ± SEM, normality was tested using the Shapiro-Wilk test, statistical analyses were performed using Unpaired *t*-test or Mann-Whitney test. Statistical significance was set at the adjusted *P*-value ≤ 0.05. **(E–G)** The “Analyse Particle” function in ImageJ was used to calculate the area and the number of IBA1^+^ cells. The same ROI defined for calculating the DG area **(E)** was used to identify the mean fluorescence intensity of the IBA1 labeling separately for each sample in the same experimental group. **(F)** The IBA1 channel was selected, filtered with the “Gamma filter” set to 1.5 and the “Gaussian Blur” set to 1 to improve image quality. **(G)** Twice the detected mean fluorescent intensity of the entire IBA1 dataset per group was used to threshold the images. For analysis the particle size was set to 8 to remove background non-specific signals. This cut-off was chosen based on resolution limitation of the fluorescent microscope. The scale bar in **(E)** also applies to **(F, G)**.

Supplementary Figure 2 Lack of correlation between KI-67 or DCX cell density and seizure parameters. In light of the non-discernible statistical disparities in cell density between the 7 and 14 dpi, as well as the absence of statistically significant correlations between total, dorsal, and ventral cell density and seizure parameters, the correlation analyses presented herein were exclusively undertaken within the subset of animals manifesting seizure activity. This was accomplished by amalgamating data from both experimental cohorts. Nonetheless, no statistical significance was observed, primarily ascribed to the limited size of the experimental group. Consequently, the data presented pertains solely to the total average density. **(A–F)** No significance was detected between KI-67 or DCX vs. Number of Seizures [**(A)** *R*^2^= 0.2512, *p* = 0.0679, **(B)** *R*^2^= 0.0163, *p* = 0.6637], Seizure Onset [**(C)** *R*^2^= 0.2082, *p* = 0.1010, **(D)** *R*^2^= 0.1103, *p* = 0.2459], or Cumulative Seizure Score [**(E)** *R*^2^= 0.2676, *p* = 0.0581, **(F)** *R*^2^= 0.0002, *p* = 0.9555]. Statistical analyses were performed using Pearson correlation coefficient test.

Supplementary Figure 3 Absence of correlation between IBA1 cell density and morphology with DCX cell density at 7 or 14 dpi. Graphs in **(A, B)** depict correlation analyses between average DCX and IBA1 density at 7 dpi (**A**, *R*^2^= 0.5665, *p* = 0.2473) or 14 dpi (**B**, *R*^2^= 0.2389, *p* = 0.5112). Concurrently, graphs in **(C, D)** show correlation analyses between average DCX cell density and average IBA1 cell area at 7 dpi (**C**, *R*^2^= 0.6880, *p* = 0.1705) or 14 dpi (**D**, *R*^2^= 0.7756, *p* = 0.1193). Statistical analyses were performed using Pearson correlation coefficient test.

Supplementary Figure 4 Regional differences in cell proliferation. **(A–P)** Analyzes of the percentage of proliferative (KI-67^+^) DCX **(A–D)**, NSC cells (NES^+^; **E**–**H**), immature astrocytes (GFAP^+^/NES^+^; **I**–**L**), or mature astrocyte (GFAP^+^; **M**–**P**) in the 14 dpi TMEV-infected C57BL/6J-mouse dorsal **(A**, **E**, **I**, **M)**, ventral **(B**, **F**, **J**, **N)**, left **(C**, **G**, **K**, **O)**, or right **(D**, **H**, **L**, **P)** hippocampus compared to CTR. **(A–D)** The significant reduction in the percentage of proliferating neuronal progenitors observed at 3 and 7 dpi, compared to CTR, was consistent across all regions [**(A)** 3 dpi vs. CTR ##*p* = 0.0078, 7 dpi vs. CTR #*p* = 0.0400, **(B)** 3 dpi vs. CTR ##*p* = 0.0041, 7 dpi vs. CTR #*p* = 0.0372, (**C**) 3 dpi vs. CTR ##*p* = 0.0048, 7 dpi vs. CTR #*p* = 0.0358, **(D)** 3 dpi vs. CTR ##*p* = 0.0056, 7 dpi vs. CTR #*p* = 0.0441]. Additionally, TMEV infection significantly reduced the percentage of proliferating NES^+^ NSC populations in all analyzed regions at 14 dpi compared to CTR [**(E)** ##*p* = 0.0048, **(F)** #*p* = 0.0159, **(G)** ##*p* = 0.0095, **(H)** ##*p* = 0.0042]. Analysis of GFAP and NES co-labeled cells showed a significant reduction compared to CTR in the left hemisphere [**(K)** #*p* = 0.0357]. Contrarily, the percentage of proliferating astrocytes was significantly higher than in CTR, in particular, in the dorsal and ventral areas [**(M)** #*p* = 0.0476, **(N)** #*p* = 0.0272]. Data are mean ± SEM, normality was tested using the Shapiro-Wilk test, statistical analyses were performed either using Kruskal-Wallis test followed by Dunn's multiple comparison test **(A–D)** or Mann-Whitney test **(E**–**M**, **P)** or Unpaired *t*-test **(N, O)**. Statistical significance was set at the adjusted (multiple comparisons) or exact *P*-value ≤ 0.05.
