# Supplementary figures and images for: Viral encephalitis and seizures cause rapid depletion of neuronal progenitor cells and alter neurogenesis in the adult mouse dentate gyrus
Source: Front Cell Neurosci. 2025 Jan 14;18:1528918. doi: 10.3389/fncel.2024.1528918 (PMC11772278; doi:10.3389/fncel.2024.1528918)

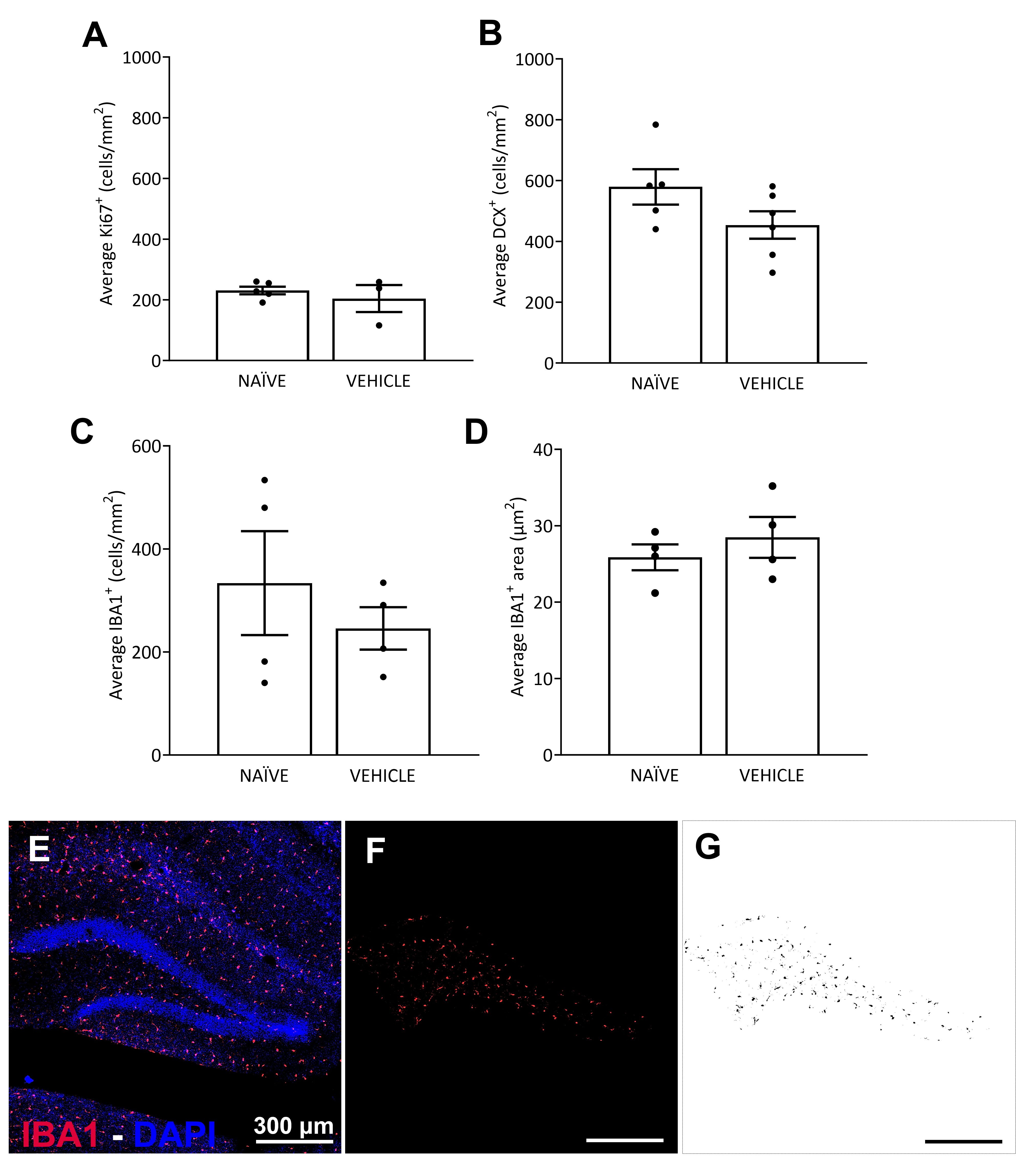

Supplement: Supplementary Figure 1 — Comparison between naïve and vehicle-injected mice and example of “Analyse Particle” analysis. (A–D) Comparative analyses between the naïve (CTR) and vehicle-injected experimental groups were performed to confirm that injection procedure itself did not influenced the analyzed parameters. No statistically significant differences were observed between naïve and vehicle-injected animals in: (A) Average KI-67+ cell density [NAÏVE (n = 5) vs. VEHICLE (n = 3) p = 0.4968], (B) Average DCX+ cell density [NAÏVE (n = 5) vs. VEHICLE (n = 6) p = 0.1172], (C) Average IBA1+ cell density [NAÏVE (n = 4) vs. VEHICLE (n = 4) p = 0.8857], and (D) Average IBA1+ Area [NAÏVE (n = 4) vs. VEHICLE (n = 4) p = 0.4434]. Data are mean ± SEM, normality was tested using the Shapiro-Wilk test, statistical analyses were performed using Unpaired t-test or Mann-Whitney test. Statistical significance was set at the adjusted P-value ≤ 0.05. (E–G) The “Analyse Particle” function in ImageJ was used to calculate the area and the number of IBA1+ cells. The same ROI defined for calculating the DG area (E) was used to identify the mean fluorescence intensity of the IBA1 labeling separately for each sample in the same experimental group. (F) The IBA1 channel was selected, filtered with the “Gamma filter” set to 1.5 and the “Gaussian Blur” set to 1 to improve image quality. (G) Twice the detected mean fluorescent intensity of the entire IBA1 dataset per group was used to threshold the images. For analysis the particle size was set to 8 to remove background non-specific signals. This cut-off was chosen based on resolution limitation of the fluorescent microscope. The scale bar in (E) also applies to (F, G). [file Image_1.TIF]

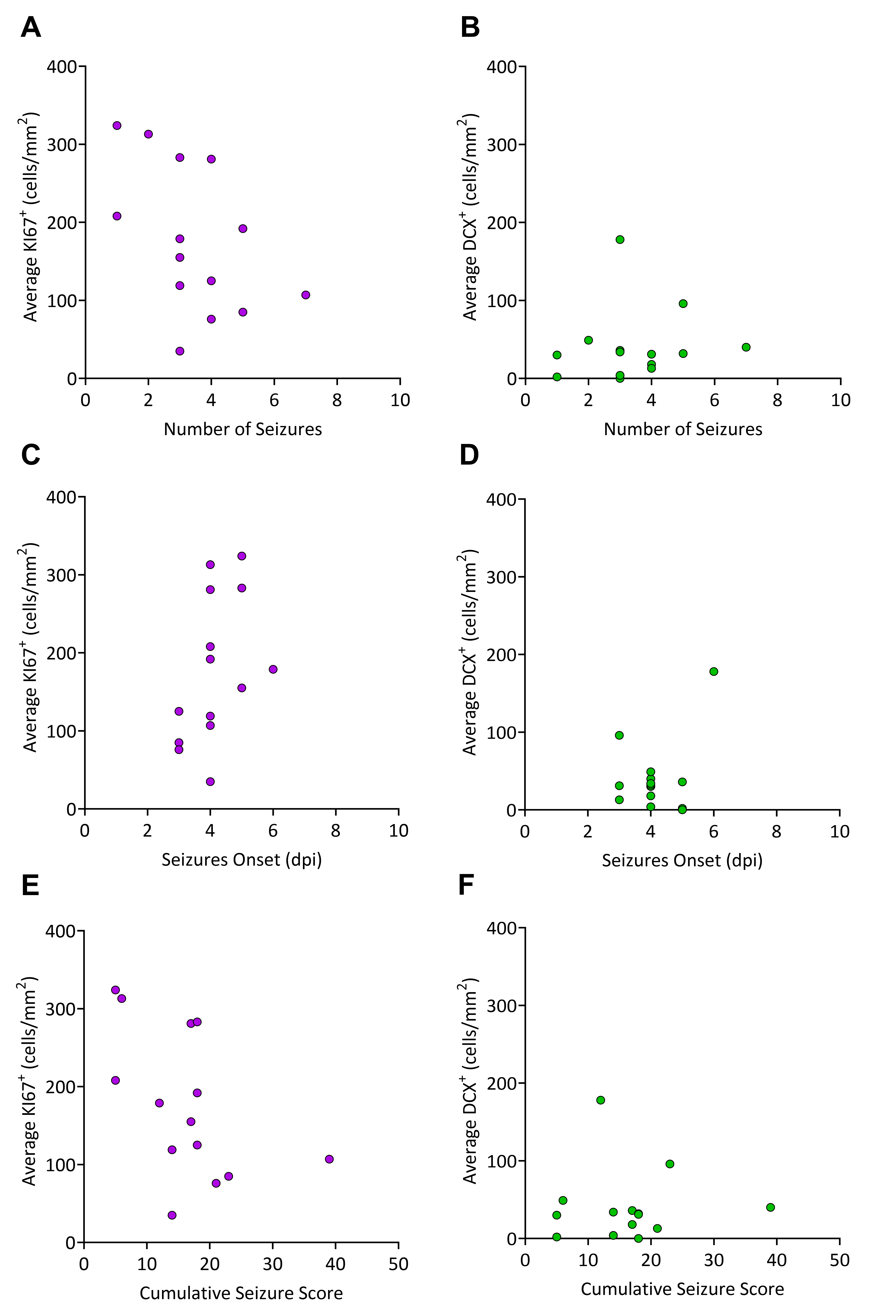

Supplement: Supplementary Figure 2 — Lack of correlation between KI-67 or DCX cell density and seizure parameters. In light of the non-discernible statistical disparities in cell density between the 7 and 14 dpi, as well as the absence of statistically significant correlations between total, dorsal, and ventral cell density and seizure parameters, the correlation analyses presented herein were exclusively undertaken within the subset of animals manifesting seizure activity. This was accomplished by amalgamating data from both experimental cohorts. Nonetheless, no statistical significance was observed, primarily ascribed to the limited size of the experimental group. Consequently, the data presented pertains solely to the total average density. (A–F) No significance was detected between KI-67 or DCX vs. Number of Seizures [(A) R2= 0.2512, p = 0.0679, (B) R2= 0.0163, p = 0.6637], Seizure Onset [(C) R2= 0.2082, p = 0.1010, (D) R2= 0.1103, p = 0.2459], or Cumulative Seizure Score [(E) R2= 0.2676, p = 0.0581, (F) R2= 0.0002, p = 0.9555]. Statistical analyses were performed using Pearson correlation coefficient test. [file Image_2.TIF]

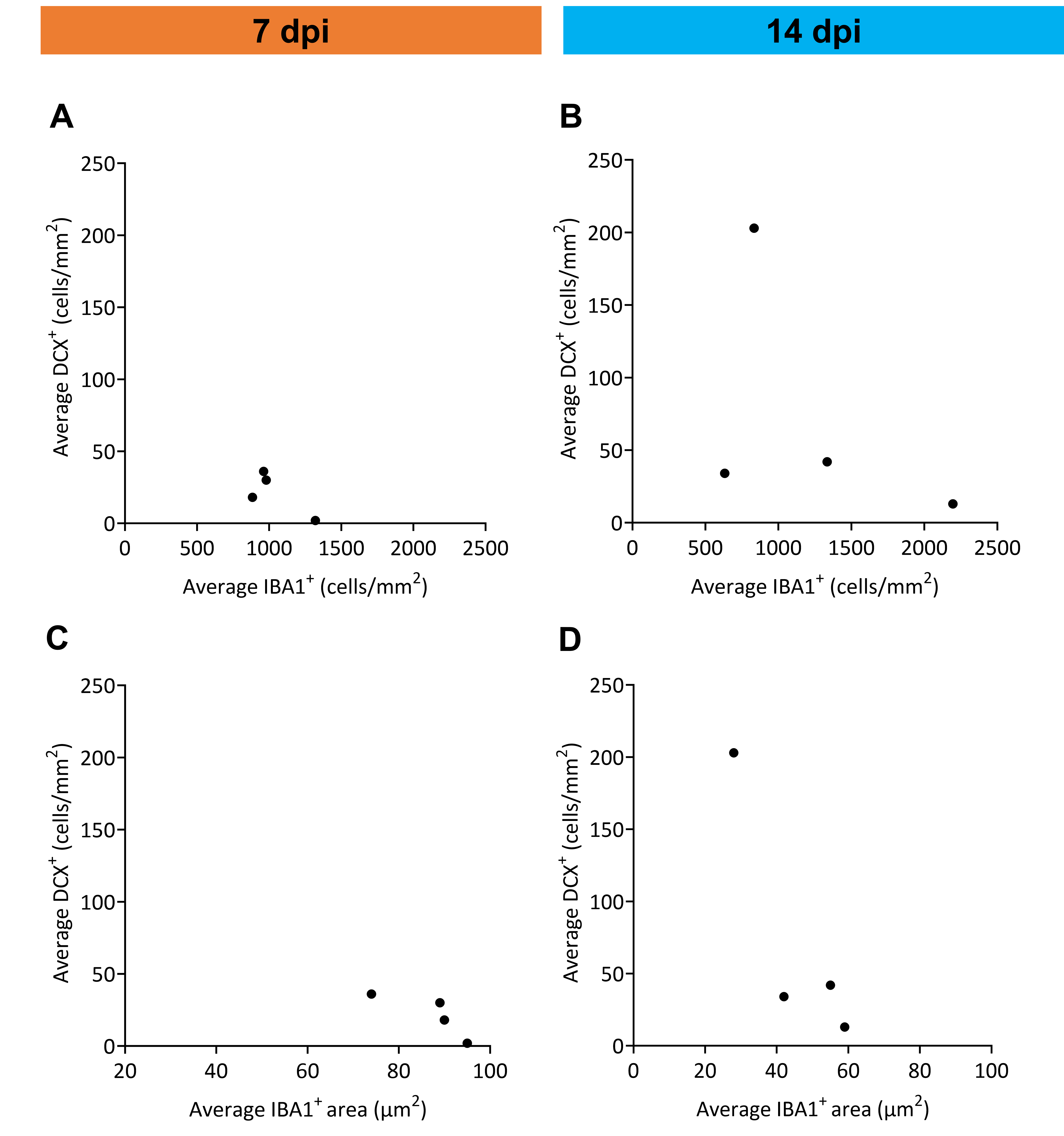

Supplement: Supplementary Figure 3 — Absence of correlation between IBA1 cell density and morphology with DCX cell density at 7 or 14 dpi. Graphs in (A, B) depict correlation analyses between average DCX and IBA1 density at 7 dpi (A, R2= 0.5665, p = 0.2473) or 14 dpi (B, R2= 0.2389, p = 0.5112). Concurrently, graphs in (C, D) show correlation analyses between average DCX cell density and average IBA1 cell area at 7 dpi (C, R2= 0.6880, p = 0.1705) or 14 dpi (D, R2= 0.7756, p = 0.1193). Statistical analyses were performed using Pearson correlation coefficient test. [file Image_3.TIF]

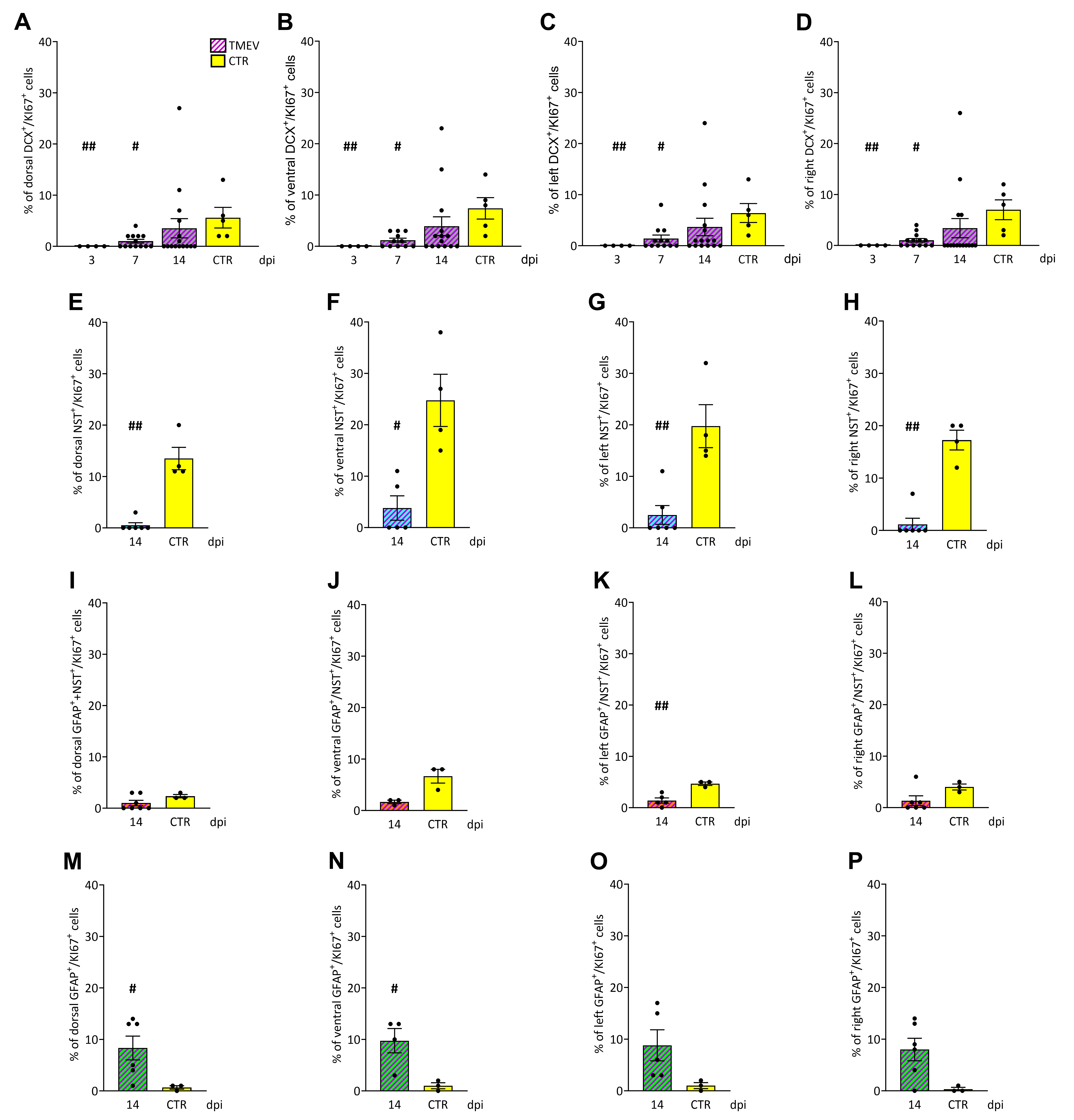

Supplement: Supplementary Figure 4 — Regional differences in cell proliferation. (A–P) Analyzes of the percentage of proliferative (KI-67+) DCX (A–D), NSC cells (NES+; E–H), immature astrocytes (GFAP+/NES+; I–L), or mature astrocyte (GFAP+; M–P) in the 14 dpi TMEV-infected C57BL/6J-mouse dorsal (A, E, I, M), ventral (B, F, J, N), left (C, G, K, O), or right (D, H, L, P) hippocampus compared to CTR. (A–D) The significant reduction in the percentage of proliferating neuronal progenitors observed at 3 and 7 dpi, compared to CTR, was consistent across all regions [(A) 3 dpi vs. CTR ##p = 0.0078, 7 dpi vs. CTR #p = 0.0400, (B) 3 dpi vs. CTR ##p = 0.0041, 7 dpi vs. CTR #p = 0.0372, (C) 3 dpi vs. CTR ##p = 0.0048, 7 dpi vs. CTR #p = 0.0358, (D) 3 dpi vs. CTR ##p = 0.0056, 7 dpi vs. CTR #p = 0.0441]. Additionally, TMEV infection significantly reduced the percentage of proliferating NES+ NSC populations in all analyzed regions at 14 dpi compared to CTR [(E) ##p = 0.0048, (F) #p = 0.0159, (G) ##p = 0.0095, (H) ##p = 0.0042]. Analysis of GFAP and NES co-labeled cells showed a significant reduction compared to CTR in the left hemisphere [(K) #p = 0.0357]. Contrarily, the percentage of proliferating astrocytes was significantly higher than in CTR, in particular, in the dorsal and ventral areas [(M) #p = 0.0476, (N) #p = 0.0272]. Data are mean ± SEM, normality was tested using the Shapiro-Wilk test, statistical analyses were performed either using Kruskal-Wallis test followed by Dunn's multiple comparison test (A–D) or Mann-Whitney test (E–M, P) or Unpaired t-test (N, O). Statistical significance was set at the adjusted (multiple comparisons) or exact P-value ≤ 0.05. [file Image_4.TIF]
